# Thermal Perceptual Thresholds are typical in Autism Spectrum Disorder but Strongly Related to Intra-individual Response Variability

**DOI:** 10.1038/s41598-019-49103-2

**Published:** 2019-08-29

**Authors:** Zachary J. Williams, Michelle D. Failla, Samona L. Davis, Brynna H. Heflin, Christian D. Okitondo, David J. Moore, Carissa J. Cascio

**Affiliations:** 1 Medical Scientist Training Program, Vanderbilt University School of Medicine, Nashville, TN 37212 USA; 2 Department of Psychiatry and Behavioral Sciences, Vanderbilt University School of Medicine, Nashville, TN 37212 USA; 3 Department of Psychology, Florida International University, Miami, FL 33199 USA; 4 Research Centre for Brain and Behaviour, Liverpool John Moores University, Liverpool, L3 5UA UK; 5 Vanderbilt Kennedy Center, Nashville, TN 37203 USA

**Keywords:** Somatosensory system, Human behaviour, Autism spectrum disorders

## Abstract

Individuals with autism spectrum disorder (ASD) are often reported to exhibit an apparent indifference to pain or temperature. Leading models suggest that this behavior is the result of elevated perceptual thresholds for thermal stimuli, but data to support these assertions are inconclusive. An alternative proposal suggests that the sensory features of ASD arise from increased intra-individual perceptual variability. In this study, we measured method-of-limits warm and cool detection thresholds in 142 individuals (83 with ASD, 59 with typical development [TD], aged 7–54 years), testing relationships with diagnostic group, demographics, and clinical measures. We also investigated the relationship between detection thresholds and a novel measure of intra-individual (trial-to-trial) threshold variability, a putative index of “perceptual noise.” This investigation found no differences in thermal detection thresholds between individuals with ASD and typical controls, despite large differences between groups in sensory reactivity questionnaires and modest group differences in intra-individual variability. Lower performance IQ, male sex, and higher intra-individual variability in threshold estimates were the most significant predictors of elevated detection thresholds. Although no psychophysical measure was significantly correlated with questionnaire measures of sensory hyporeactivity, large intra-individual variability may partially explain the elevated psychophysical thresholds seen in a subset of the ASD population.

## Introduction

Autism spectrum disorder (ASD) is a complex, heterogeneous neuropsychiatric syndrome characterized by difficulties with social communication and the presence of repetitive and stereotyped interests and behaviors^1^. In addition, with the publication of the fifth edition of the *Diagnostic and Statistical Manual of Mental Disorders* (DSM–5^1^), the diagnostic criteria for ASD have been updated to include “Hyper- or hyporeactivity to sensory input or unusual interest in sensory aspects of the environment.” While much recent ASD literature has sought to address the underpinnings of sensory hyperreactivity exhibited by individuals with ASD^2–5^, much less is known about sensory hyporeactivity in this population. Perhaps the most cited example of hyporeactivity in ASD is that these individuals exhibit an “apparent indifference to pain/temperature”^1^. This lack of response to ostensibly noxious stimuli has led many investigators to infer that individuals with ASD exhibit reduced or absent perception of these stimuli^6^. However, evidence suggesting a reduction in pain and temperature perception in ASD has predominantly been inferred from clinical case reports, parent interviews, and questionnaire studies^7,8^, and recent reviews question the long-held assumption that individuals with ASD truly are hyposensitive in these modalities^7,9,10^.

Leading models of sensory hyporeactivity^11–14^ postulate that observed indifference to stimuli is the result of elevated thresholds for stimulus perception. Perceptual thresholds are defined by the minimum amount of stimulus energy necessary to register the percept. For thermal stimuli, the stimulus energy is a temperature change from a baseline temperature. The direction of this change is thus opposite for warmth and cool detection thresholds (i.e., higher temperatures for warmth detection and lower temperatures for cool detection both correspond to elevated perceptual thresholds. In this report, we will refer to warm and cool thresholds in terms of degree of change from baseline temperature rather than absolute temperature to maximize consistency and clarity. These proposed mechanisms for aberrant sensory responsivity are appropriately tested using the methods of experimental psychophysics to relate objective stimulus intensity to perceptual threshold^15^. Psychophysical warm and cold thermal detection thresholds have been widely studied in both healthy and clinical populations (for a review, see^16^), and these measures allow researchers to empirically test the presupposed group differences in stimulus perception.

Relatively few studies have used psychophysical methods to study hyposensitivity in the ASD population, and most studies to date have focused on differences in pain thresholds. Intriguingly, psychophysical studies commonly report no significant differences in pain thresholds between individuals with ASD and typically-developing (TD) controls^17–23^, and several have even reported *reduced* pain thresholds in the ASD group^24–27^. Of particular interest, no study to date has found that psychophysical pain thresholds are substantially higher in ASD than in TD controls. Even fewer studies have assessed detection thresholds for nonpainful warm and cold stimuli, with mixed results. Three have found no difference in warm or cool detection thresholds between individuals with ASD and TD controls^20,23,24^, while Duerden *et al*.^18^ reported that the ASD group exhibited higher warm and cool detection thresholds (i.e., a larger change from baseline was needed for detection in both conditions). The ASD group in the study by Yasuda and colleagues^22^ demonstrated lower cold detection thresholds (i.e., detecting cold at a lower change from baseline) compared to controls, although no difference was found in warm detection thresholds. These studies all have important limitations, including small sample sizes (≤20 ASD participants) and the failure to account for confounding variables such as age, sex, and IQ, which often differed between ASD and control groups.

The largest of these studies, conducted by Duerden and colleagues^18^, compared method-of-limits thermal detection and pain thresholds between a group of adolescents with ASD and IQ > 70 (n = 20) and TD controls (n = 55) who were administered the same psychophysical task in a separate study. As noted above, the ASD group reported significantly higher thresholds for both warmth and cold detection, consistent with reduced sensitivity to thermal stimuli of both types. In addition, detection thresholds in the ASD group were strongly correlated with IQ (*r*_warm_ = −0.8, *r*_cold_ = 0.59), suggesting that lower IQ is associated with apparent hyposensitivity to both heat and cold. However, because IQ scores were not measured in the TD group, the apparent group differences may have resulted from unmeasured differences in IQ between groups rather than differences in ASD diagnostic status *per se*. Notably, a later study that explicitly matched ASD and TD groups on verbal IQ reported no significant differences in detection thresholds across groups^20^.

In addition to the above limitations, studies to date have not taken into account the distributional properties of thermal thresholds. Multiple large-studies have demonstrated that thermal thresholds in the general population are non-normally distributed, typically due to high levels of skewness^28–31^. Because of this skewness, ASD-TD threshold comparisons using Student’s *t* test and similar procedures will result in an unacceptably high type I error rate at the sample sizes typically encountered in psychological research^32,33^. However, such limitations are easily overcome by instead conducting group comparisons using nonparametric statistics such as the Wilcoxon-Mann-Whitney test^29,30^, which maintain their nominal type I error rate under a much wider range of distributional conditions^33^.

The current study tested the hypothesis that individuals with ASD exhibit significantly elevated warm and cool detection thresholds consistent with behavioral reports of reduced reactivity to thermal stimuli. This study specifically addresses the methodological limitations of past research in this area, employing a relatively large sample with a wide age range, accounting for a number of potentially confounding variables, and employing rank-based statistical methods that are robust to the non-normality inherent in psychophysical threshold data. We also extended the work of prior authors by examining the degree to which psychophysical thresholds are associated with individual differences in age, sex, IQ, and common measures of autism symptomatology (ADOS-2 Calibrated Severity Score [CSS]^34–36^, Social Responsiveness Scale – Second Edition [SRS-2]^37^), including sensory features (Adolescent/Adult Sensory Profile [AASP]^38,39^ and Sensory Profile [SP]^40^ quadrant scores, as well as a one-item measure of sensory hyperresponsivity derived from the SRS-2). Lastly, as some studies have linked ASD to increasingly noisy and variable perceptions^41–44^, we investigated diagnostic group differences in intra-individual variability in warm and cool threshold temperatures across individual trials of our experimental task (as measured by Gini’s Mean Difference [*GMD*]^45–47^), as well as the relationships between *GMD* values and detection thresholds derived from those same trials.

## Results

### Descriptive statistics and group comparisons

In total, 142 participants were included in the final study sample: 32 adults with ASD (21 male, median age 25.50 years), 24 adults with typical development (TD) (14 male, median age 29.76 years), 51 children and adolescents with ASD (41 male, median age 10.03 years), and 35 children and adolescents with TD (26 male, mean age 9.21 years), ages 7.0–17.99 years (Table 1).

**Table 1:** Participant demographics by diagnostic group.

	ASD	TD	Whole Sample
Age group (Adult/Child)	32/51	24/35	56/86
Sex (M/F)	62/21	40/19	102/40
Race			
White	52	32	84
Black/African-American	3	6	9
Asian-American	3	3	6
American Indian/Alaska Native	1	1	2
Mixed race	6	0	6
NA/Prefer not to respond	16	16	32
Hispanic ethnicity	4	1	5
Annual household income (USD)			
<$20,000	11	4	15
$20,000–$40,000	10	8	18
$40,000–$60,000	9	5	14
$60,000–$80,000	9	5	14
$80,000–$100,000	9	4	13
>$100,000	17	17	34
NA/Prefer not to respond	18	16	34
Psychiatric medication (ASD only)			
Any psychiatric medication	26	NA	26
SSRI	9	NA	9
Psychostimulant	11	NA	11
Other^†^	12	NA	12

^†^Includes alpha-2 agonists (n = 4), benzodiazepines (n = 3), atypical antipsychotics (n = 3), hydroxyzine (n = 1), mirtazapine (n = 1), zolpidem (n = 1), oxcarbazepine (n = 1).

ASD-TD group differences were tested using Cliff’s delta (δ)^48–50^, a non-parametric test statistic that doubles as a standardized effect size (ranging from −1 to 1). Furthermore, we supplemented null-hypothesis significance tests of group differences with equivalence testing^51,52^, which allows us to test the null hypothesis that the difference between two groups is greater than or equal to a pre-specified smallest effect size of interest (in this case, an effect of “medium” magnitude). If the equivalence test null hypothesis is rejected, it provides statistical evidence that group differences are smaller than the smallest effect size of interest (“statistically equivalent”) and are thereby too small to be theoretically meaningful. Thus, hypotheses of group differences can be interpreted in one of four ways: (a) statistically different from zero and not statistically equivalent (*p* < 0.05, *p*_equiv_ > 0.05), (b) not statistically different from zero and statistically equivalent (*p* > 0.05, *p*_equiv < _0.05) (c) statistically different from zero and statistically equivalent, i.e., a nonzero effect that is significantly smaller than the smallest effect size of interest (*p* < 0.05, *p*_equiv < _0.05), or (d) not statistically different from zero and not statistically equivalent, i.e., inconclusive (*p* > 0.05, *p*_equiv_ > 0.05)^51^. Summary statistics and group comparisons are available in Table 2.

**Table 2:** Descriptive statistics and diagnostic group comparisons.

Variable	N (ASD/TD)	ASD [*Mdn*, (Q_1_, Q_3_)]	TD [*Mdn*, (Q_1_, Q_3_)]	δ (90% CI)	*P*_*H0*_	*P*_*equiv*_
Age (Years)	83/59	15.27 (9.28, 22.7)	13.26 (8.83, 27.84)	0.014 (−0.154, 0.180)	0.894	**0.001**
Adults	32/24	25.5 (21.13, 33.61)	29.76 (25.55, 33.29)	−0.259 (−0.487, 0.002)	0.103	0.316
Children	51/35	10.03 (8.47, 14.05)	9.21 (8.20, 10.86)	0.207 (−0.001, 0.398)	0.102	0.151
Verbal IQ	80/58	100.58 (89.58, 110.68)	108.85 (99.51, 119.6)	−0.347 (−0.487, −0.191)	**<0.001**	0.576
Adults	30/23	101.14 (93.14, 110.74)	105.81 (98.24, 116.98)	−0.265 (−0.498, 0.003)	0.103	0.335
Children	50/35	100.21 (87.21, 110.97)	110.74 (101.04, 121.88)	−0.399 (−0.566, −0.200)	**0.002**	0.725
Performance IQ	80/58	105.15 (95.45, 117.27)	109.18 (97.71, 122.62)	−0.122 (−0.282, 0.044)	0.224	**0.015**
Adults	30/23	107.98 (95.57, 116.04)	106.26 (96.46, 116.41)	0.009 (−0.254, 0.271)	0.957	**0.021**
Children	50/35	103.47 (95.1, 118.8)	114.01 (98.08, 124.54)	−0.191 (−0.39, 0.024)	0.143	0.130
Full-scale IQ	80/58	103.86 (93.15, 113.55)	111.66 (101.85, 120.35)	−0.285 (−0.433, −0.123)	**0.005**	0.316
Adults	30/23	104.61 (96.11, 112.93)	107.44 (98.54, 117.3)	−0.116 (−0.369, 0.154)	0.480	0.085
Children	50/35	103.29 (91.38, 114.39)	114.16 (104.15, 121.9)	−0.373 (−0.548, −0.167)	**0.004**	0.642
SRS-2 Total T-score	71/40	71.27 (64.18, 78.96)	42.36 (39.51, 48.7)	0.971 (0.935, 0.988)	**<0.001**	>0.999
Adults	26/18	68.58 (60.46, 77.76)	43.28 (40.09, 49.65)	0.942 (0.841, 0.980)	**<0.001**	>0.999
Children	45/22	73.42 (65.7, 79.69)	42.06 (39.17, 47.9)	0.988 (0.959, 0.996)	**<0.001**	>0.999
SRS-2 Item 42 (0–3)	72/47	2.02 (1.02, 2.99)	0.00 (0.00, 0.86)	0.729 (0.611, 0.815)	**<0.001**	>0.999
Adults	26/18	2.65 (1.72, 3.00)	0.17 (0.00, 0.97)	0.771 (0.560, 0.888)	**<0.001**	0.998
Children	46/29	1.73 (0.99, 2.81)	0.01 (0.00, 0.65)	0.724 (0.570, 0.829)	**<0.001**	>0.999
AASP Scores - Adults						
Low Registration	24/20	41.74 (34.39, 50.18)	24.49 (22.68, 28.21)	0.869 (0.672, 0.951)	**<0.001**	>0.999
Sensory Seeking	24/20	37.25 (33.71, 41.64)	45.42 (39.67, 49.2)	−0.554 (−0.746, −0.278)	**0.002**	0.914
Sensory Sensitivity	24/20	48.96 (39.88, 54.92)	33.39 (31.21, 37.11)	0.748 (0.501, 0.882)	**<0.001**	0.994
Sensory Avoiding	24/20	49.98 (40.99, 58.32)	32.17 (27.94, 38.59)	0.733 (0.489, 0.871)	**<0.001**	0.993
SP Scores - Children						
Low Registration	44/30	55.86 (47.04, 62.15)	70.06 (66.49, 72.66)	−0.861 (−0.929, −0.735)	**<0.001**	>0.999
Sensory Seeking	44/30	94.22 (86.26, 106.14)	110.94 (104.04, 119.69)	−0.58 (−0.732, −0.375)	**<0.001**	0.975
Sensory Sensitivity	44/30	69.66 (60.48, 82.12)	88.67 (81.53, 94.83)	−0.734 (−0.839, −0.576)	**<0.001**	>0.999
Sensory Avoiding	44/30	99.82 (90.28, 111.09)	122.85 (114.63, 130.92)	−0.817 (−0.897, −0.685)	**<0.001**	>0.999
ADOS-2 Overall CSS	72/0	7.95 (6.20, 9.41)	N/A	N/A	N/A	N/A
Adults	29/0	7.34 (6.04, 8.95)	N/A	N/A	N/A	N/A
Children	43/0	8.63 (6.59, 9.56)	N/A	N/A	N/A	N/A
Warm Threshold (°C)	83/59	1.88 (1.25, 2.87)	1.56 (1.12, 2.36)	0.171 (0.009, 0.323)	0.078	**0.043**
Adults	32/24	1.77 (1.34, 2.89)	1.33 (0.97, 2.11)	0.339 (0.074, 0.559)	**0.027**	0.512
Children	51/35	2.02 (1.20, 2.87)	1.74 (1.25, 2.54)	0.072 (−0.135, 0.273)	0.567	**0.016**
Cool Threshold (°C)	83/59	2.41 (1.83, 3.47)	2.18 (1.75, 2.72)	0.178 (0.019, 0.328)	0.065	**0.046**
Adults	32/24	2.20 (1.76, 3.43)	1.95 (1.61, 2.54)	0.201 (−0.058, 0.434)	0.192	0.190
Children	51/35	2.62 (1.90, 3.55)	2.29 (1.88, 2.85)	0.159 (−0.049, 0.353)	0.201	0.074
Warm GMD (°C)	83/59	0.46 (0.29, 0.78)	0.61 (0.36, 0.98)	0.195 (0.034, 0.347)	**0.043**	0.073
Adults	32/24	0.30 (0.21, 0.48)	0.41 (0.26, 0.76)	0.250 (−0.008, 0.477)	0.100	0.293
Children	51/35	0.60 (0.41, 0.89)	0.71 (0.47, 1.19)	0.159 (−0.05, 0.355)	0.205	0.077
Cool GMD (°C)	83/59	0.40 (0.25, 0.67)	0.56 (0.34, 1.11)	0.248 (0.085, 0.397)	**0.010**	0.189
Adults	32/24	0.28 (0.17, 0.49)	0.45 (0.26, 0.81)	0.337 (0.076, 0.555)	**0.026**	0.519
Children	51/35	0.47 (0.32, 0.86)	0.62 (0.41, 1.28)	0.206 (−0.007, 0.401)	0.104	0.153

*Note*. Threshold values indicate changes (in °C) from the baseline temperature of 32 °C. δ = Cliff’s (1993) delta statistic (an effect size metric); Q_1_ = first quartile; Q_3_ = third quartile; *P*_*H0*_ = *p*-value for test of null hypothesis of no effect; *P*_*equiv*_ = *p*-value for equivalence test (H_0_: |δ| ≥ 0.33, H_A_: |δ|**<**0.33); AASP = Adolescent/Adult Sensory Profile; *GMD* = Gini’s Mean Difference; SP = Sensory Profile; SRS-2 = Social Responsiveness Scale – Second Edition; SRS-2 item 42 = “*I am overly sensitive to certain sounds, textures, or smells”* (self-report) or “*Seems overly sensitive to certain sounds, textures, or smells”* (caregiver report).

The ASD and TD groups were statistically equivalent in terms of age, δ = 0.014, *p* = 0.894, *p*_equiv_ = 0.001, and performance IQ (PIQ), δ = −0.122, *p* = 0.224, *p*_equiv_ = 0.015, and there were no significant group differences in sex ratio, χ^2^(1) = 0.81, *p* = 0.368, Φ = 0.076. The ASD group did have significantly lower verbal IQ (VIQ), δ = −0.347, *p* < 0.001, *p*_equiv_ = 0.576, and full-scale IQ (FSIQ) scores, δ = −0.285, *p* = 0.005, *p*_equiv_ = 0.316. These patterns were similar when analyses were restricted to either the child/adolescent or adult groups (Table 2), with the differences in VIQ and FSIQ no longer significant in the adult subsample.

As expected, large and significant group differences were seen in all questionnaire measures of ASD traits and sensory features (Table 2). SRS-2 T-scores were substantially higher in the ASD group (*Mdn* = 71.27, *IQR* [64.18, 78.96]) compared to the TD group (*Mdn* = 42.36, *IQR* [39.51, 48.70]), δ = 0.971, *p* < 0.001. These group differences were similarly strong for both adult self-reports, δ = 0.942, *p* < 0.001, and caregiver reports, δ = 0.988, *p* < 0.001, when considered separately. Scores on the SRS-2 one-item sensory question were also substantially elevated in the ASD group, δ = 0.729, *p* < 0.001, with 64% of the ASD group endorsing values of 3 (*often true*) or 4 (*always true*) compared to only 6% of the TD group. Large group differences were also found on all SP and AASP quadrant scores, all |δ| > 0.554, *p*s < 0.001, *p*s_equiv_ > 0.914, with both ASD age groups exhibiting elevated scores in the domains of low registration, sensory sensitivity, and sensory avoiding. The ASD group showed divergent results for sensory seeking on the two sensory questionnaires, with higher levels of sensory seeking reported on the caregiver SP, δ = −0.580, *p* < 0.001 (note that lower scores on the caregiver SP represent higher levels of the reported behavior), as well as reduced sensory-seeking reported on the self-report AASP, δ = −0.554, *p* < 0.001.

**Figure 1:**
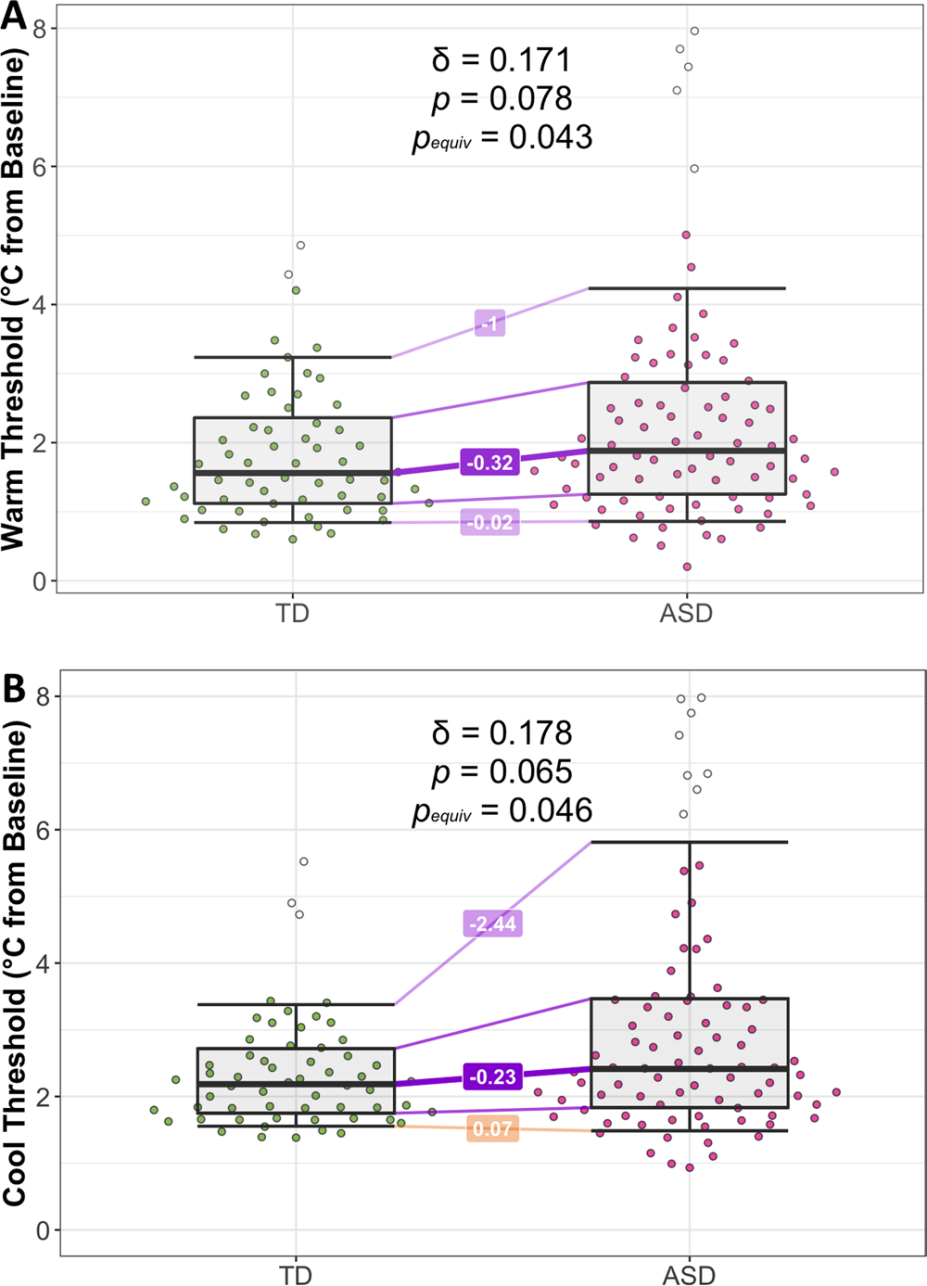
Comparison of thermal detection thresholds in the two diagnostic groups. (**A**) Warm trial median threshold values based on *n* = 10 trials per subject. (**B**) Cool trial median threshold values based on *n* = 10 trials per subject. Horizontal lines are not typical boxplot marks but instead represent the 0.1, 0.25, 0.5, 0.75, and 0.9 Harrell-Davis quantiles of each group’s distribution. Differences in group quantiles (TD – ASD) are depicted as lines bridging the two groups. Outliers (defined by applying the boxplot rule to each group distribution) are represented as unshaded points.

Warm and cool detection thresholds, as well as the respective *GMD* values from those trials, were compared between the ASD and TD groups in the entire sample, as well as the child/adolescent and adult subsamples separately (Table 2). In the overall sample, the groups were equivalent in terms of warm detection threshold, δ = 0.171, *p* = 0.078, *p*_equiv_ = 0.043, and cool detection threshold, δ = 0.178, *p* = 0.065, *p*_equiv_ = 0.046. When considering the child/adolescent subsample alone, the group differences were not statistically different but only equivalent in terms of warm threshold (Table 2). However, when analyses were restricted to the adult group, the ASD group exhibited significantly higher thresholds for warmth detection, δ = 0.339, *p* = 0.037, *p*_equiv_ = 0.523. Cool thresholds were higher in adults with ASD than their TD counterparts, but this difference was neither statistically significant nor equivalent, δ = −0.208, *p* = 0.185, *p*_equiv_ = 0.205. Although most threshold differences were small and non-significant, the ASD group consistently demonstrated higher warm and cool thresholds (i.e., higher warm threshold temperatures and colder cool threshold temperatures). Upon visual inspection of the data, these trends seemed to be due to a small number of outlying values (defined by the boxplot rule^53^) in the ASD group rather than a difference across all quantiles of the threshold distribution (Fig. 1).

*GMD* values in the ASD group were significantly larger than the TD group in both conditions, but effect sizes were small, Warm: δ = 0.195, *p* = 0.043, *p*_equiv_ = 0.073, Cool: δ = 0.248, *p* = 0.010, *p*_equiv_ = 0.189. The *GMD* distributions in both ASD and TD groups contained a substantial number of outliers (approximately 5–10% of each group; Supplementary Fig. S1), without significant differences in outlier proportions between the two diagnostic groups, Fisher’s exact tests: *p*s > 0.362.

In summary, the ASD and TD groups did not differ in terms of age, sex, and PIQ, though the TD group did exhibit higher VIQ and FSIQ scores. Consistent with expectations, the ASD group had elevated scores on measures of autistic traits and sensory features in the domains of low registration, sensory sensitivity, and sensory avoiding. Scores for sensory seeking differed between diagnostic groups, but the directionality of the effect differed in children and adults. Neither warm nor cool detection thresholds were significantly different between groups, and statistical equivalence tests were significant for both modalities in the whole sample. Despite equivalent threshold measurements, the ASD group did exhibit a small but significant increase in trial-to-trial variability across conditions.

### Correlational analyses

**Figure 2:**
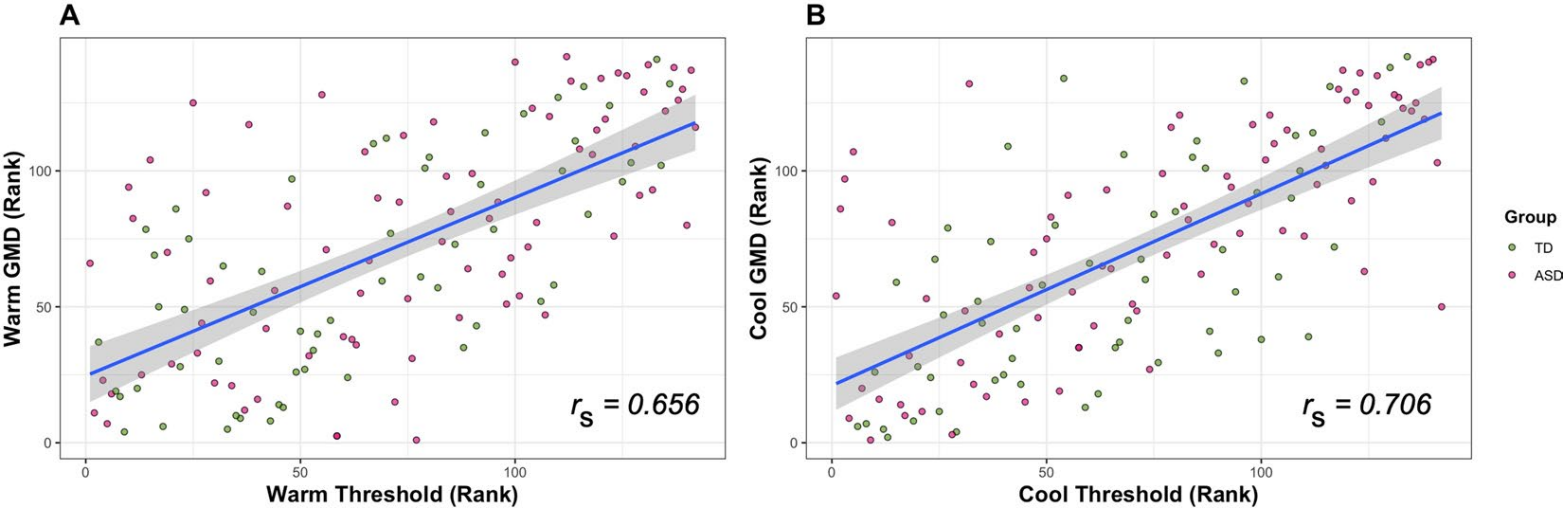
Spearman rank correlations between thresholds and intra-individual variability (GMD). Scatterplot displaying the rank correlation between warm (**A**) and cool (**B**) detection threshold values and individual *GMD* values. Threshold and *GMD* values are based on *n* = 10 trials per modality per subject. Correlations are approximately equal when considering the ASD and TD groups separately.

Relationships between thermal detection thresholds and additional predictor variables (i.e., *GMD* values, age, IQ scores, ASD symptoms, and self- or caregiver-reported sensory abnormalities) were assessed using Spearman rank correlations, with equivalence tests performed to indicate which correlations were significantly smaller than the minimum correlation of interest (H_A_: |*r*_s_| < 0.3). Warm and cool detection threshold temperatures were highly correlated across the whole sample, *r*_s_ = 0.840, *p* < 0.001, as were the *GMD* values from warm and cool trials, *r*_s_ = 0.603, *p* < 0.001. Large correlations were also found between detection threshold and the *GMD* from the trials in each modality, Warm: *r*_s_ = 0.656, *p* < 0.001; Cool: *r*_s_ = 0.706, *p* < 0.001 (Fig. 2). The values of the above correlations were not significantly different when comparing the ASD and TD subsamples (all |*r*_ASD_ – *r*_TD_| < 0.132, all 95% CIs included zero).

Warm detection threshold was significantly correlated with PIQ, *r*_s_ = −0.237, *p* = 0.006, *p*_equiv_ = 0.217, but not VIQ, *r*_s_ = −0.039, *p* = 0.654, *p*_equiv_ = 0.001, and the difference between the two correlations was also statistically significant, *r*_piq_ − *r*_viq_ = −0.198, 95% CI [−0.360, −0.032]. Cool detection threshold also exhibited a significant correlation with PIQ, *r*_s_ = −0.322, *p* < 0.001, no significant correlation with VIQ, *r*_s_ = −0.110, *p* = 0.200, and a significant difference between the two correlations, *r*_piq_ − *r*_viq_ = −0.212, 95% CI [−0.372, −0.049]. These associations between PIQ and detection thresholds were also not significantly different in the ASD and TD groups (Warm: *r*_ASD_ − *r*_TD_ = −0.193, 95% CI [−0.512, 0.131]; Cool: *r*_ASD_ − *r*_TD_ = −0.159, 95% CI [−0.469, 0.148]). Age was not significantly associated with warm detection threshold, *r*_s_ = −0.080, *p* = 0.345, *p*_equiv_ = 0.004, although a significant association with cool detection threshold did emerge, *r*_s_ = −0.176, *p* = 0.038, *p*_equiv_ = 0.062. However, the difference between the correlations of age with warm and cool thresholds was not statistically significant, *r*_cool_ – *r*_warm_ = −0.096, 95% CI [−0.188–0.003]. There were no significant associations between either detection threshold and SRS-2 T-score, SRS-2 sensory item score, SP sensory item score, SP quadrant scores, or AASP quadrant scores, all |*r*_s_| < 0.148, *p*s > 0.223, with the majority of these correlations falling within the equivalence bounds (Supplementary Tables S2–S4). Similarly, warm and cool *GMD* values were not significantly correlated with either SP or AASP scores, and many of these correlations were found to be statistically equivalent (Supplementary Tables S3 and S4). In the ASD group, ADOS-2 CSS was not significantly correlated with warm detection threshold, *r*_s_ = 0.151, *p* = 0.209, *p*_equiv_ = 0.665, although it was correlated with cool detection threshold, *r*_s_ = 0.281, *p* = 0.018, *p*_equiv_ = 0.938. The difference between these two correlations was statistically significant, *r*_cool_ – *r*_warm_ = 0.130, 95% CI [2.39 × 10^−5^, 0.260]. The full matrix of Spearman correlations can be found in the Supplementary Tables S2–S4.

These analyses indicate that warm and cool detection thresholds were highly correlated, displaying similar patterns of association with other variables. Increased threshold was strongly related to both lower PIQ and higher *GMD*. These correlations were not significantly different between the diagnostic groups. None of the other covariates were significantly correlated with thermal detection thresholds across the entire sample, and many were found to be statistically equivalent (Supplementary Table S2). When considering the ASD group alone, higher ADOS-2 CSS values were related to higher cool but not warm detection thresholds.

### Regression models

To assess group differences in thermal detection thresholds while controlling for other variables, we conducted hierarchical multiple regressions using a robust semi-parametric proportional odds model^54,55^. Three sequential models were fit for each detection threshold, with predictors that consisted of: (1) diagnosis, age, sex, and counterbalance order, (2) model 1 plus additional variables based on a best-subset regression analysis (see Methods for more details), and (3) model 2 plus the corresponding *GMD* value (Table 3).

**Table 3:** Regression models for warm and cool detection thresholds across the entire sample.

Warm Step 1: Baseline Model		Wald χ ^2^	*P*	Cool Step 1: Baseline Model		Wald χ ^2^	*P*
Predictor	aOR (95% CI)			Predictor	aOR (95% CI)		
Diagnosis (ASD)	1.60 (0.89, 2.86)	2.49	0.114	Diagnosis (ASD)	1.67 (0.93 2.98)	3.00	0.083
Sex (Male)	1.90 (0.98, 3.70)	3.60	0.058	Sex (Male)	1.71 (0.88, 3.31)	2.53	0.112
Age (Years)	1.00 (0.97, 1.02)	0.08	0.774	Age (Years)	0.98 (0.95, 1.01)	1.63	0.202
Counterbalance	1.26 (0.71, 2.24)	0.63	0.429	Counterbalance	1.35 (0.76, 2.40)	1.05	0.305
**Model Fit**	χ^2^(4) = 7.58	*p* = 0.108	*R*^2^ = 0.052	**Model Fit**	χ^2^(4) = 9.61	*p* = 0.0475*	*R*^2^ = 0.065
**Warm Step 2: Best-subset Regression Model**				**Cool Step 2: Best-subset Regression Model**			
**Predictor**	**aOR (95% CI)**	**Wald χ** ^**2**^	***P***	**Predictor**	**aOR (95% CI)**	**Wald χ** ^**2**^	***P***
Diagnosis (ASD)	1.43 (0.80, 2.57)	1.44	0.230	Diagnosis (ASD)	1.41 (0.78, 2.52)	1.30	0.254
**Sex (Male)**	**2.41 (1.23, 4.74)**	**6.56**	0.010*	**Sex (Male)**	**2.30 (1.17, 3.54)**	**5.84**	0.016*
Age (Years)	1.00 (0.97, 1.03)	**<**0.01	0.974	Age (Years)	0.98 (0.95, 1.01)	1.48	0.223
Counterbalance	1.29 (0.73, 2.29)	0.75	0.386	Counterbalance	1.38 (0.77, 2.46)	1.29	0.276
**PIQ**	**0.97 (0.96, 0.99)**	**8.97**	0.003*	**PIQ**	**0.96 (0.95, 0.98)**	**15.42**	<0.001*
**Model Fit**	χ^2^(5) = 16.64	*p* = 0.005*	*R*^2^ = 0.111	**Model Fit**	χ^2^(5) = 25.49	*p***<**0.001*	*R*^2^ = 0.164
**Warm Step 3: Best-subset Model with** ***GMD***				**Cool Step 3: Best-subset Model with** ***GMD***			
**Predictor**	**aOR (95% CI)**	**Wald χ** ^**2**^	***P***	**Predictor**	**aOR (95% CI)**	**Wald χ** ^**2**^	***P***
Diagnosis (ASD)	1.10 (0.61, 1.99)	0.11	0.741	Diagnosis (ASD)	1.29 (0.72, 2.32)	0.75	0.385
**Sex (Male)**	**2.23 (1.15, 4.35)**	**5.57**	0.018*	**Sex (Male)**	**2.54 (1.30, 4.98)**	**7.38**	**0.007**
**Age (Years)**	**1.03 (1.00, 1.06)**	**4.68**	0.030*	Age (Years)	1.01 (0.98, 1.04)	0.33	0.568
Counterbalance	1.30 (0.73, 2.32)	0.78	0.377	Counterbalance	1.29 (0.73, 2.28)	0.76	0.384
PIQ	0.99 (0.97, 1.01)	1.46	0.227	**PIQ**	**0.98 (0.96, 0.99)**	**7.37**	0.007*
**Warm** ***GMD***	**11.88 (5.94, 23.80)**	**48.82**	<0.001*	**Cool** ***GMD***	**5.79 (3.51, 9.54)**	**47.40**	<0.001*
**Model Fit**	χ^2^(6) = 73.53	*p* < 0.001*	*R*^2^ = 0.404	**Model Fit**	χ^2^(6) = 86.83	*p* < 0.001*	*R*^2^ = 0.457

*Note*. Significant predictors in each model are bolded; PIQ = Performance IQ; *GMD* = Gini’s Mean Difference.

*p < 0.05.

The baseline model for warm detection threshold was not significantly better than the intercept-only null model, χ^2^(4) = 7.58, *p* = 0.108, Nagelkerke^56^ *R*^2^ = 0.052; ASD diagnosis, age, sex and counterbalance order all failed to significantly predict warm detection threshold (Table 3). In the best-subset regression analysis, the baseline model plus PIQ was chosen as the best model, BIC weight = 0.490, Evidence Ratio vs. baseline model = 10.2 (information on competing models and predictor BIC weights can be found in Supplementary Information). The second model fit significantly better than baseline, χ^2^(1) = 9.06, *p* = 0.003, ∆*R*^2^ = 0.059, and PIQ was found to be a strong predictor of threshold. Individuals with higher IQ scores tended to report lower warmth detection thresholds. After adding PIQ to the model, sex was also a significant predictor, with males reporting significantly higher detection thresholds than females. The addition of the warm *GMD* to the model in the next step resulted in a substantial improvement in fit, χ^2^(1) = 56.89, *p* < 0.001, ∆*R*^2^ = 0.294. Warm trial *GMD* was a highly significant predictor of warm detection threshold, with higher *GMD* values predicting elevated thresholds. After the addition of *GMD* to the model, PIQ was no longer a significant predictor of warm detection threshold, although the effect of sex remained significant. In this model, age was also a significant predictor, with older age related to higher warm detection thresholds after controlling for intra-individual variability.

A similar pattern of results was seen for the cool detection threshold models (Table 3). The baseline model did fit significantly better than the null model, χ^2^(4) = 9.61, *p* = 0.057, Nagelkerke *R*^2^ = 0.065, although no predictors were significant. In the best-subset regression analysis, the baseline model plus PIQ was again the best model, BIC weight = 0.557, Evidence Ratio vs. baseline model = 353.3, and this model exhibited significantly better fit to the data than the baseline model, χ^2^(1) = 15.87, *p* < 0.001, ∆*R*^2^ = 0.099. As with the warm detection threshold, cool detection threshold was predicted by sex and PIQ, with males and individuals with lower PIQ scores exhibiting higher cool detection thresholds. Adding *GMD* to the model improved fit further, χ^2^(1) = 61.34, *p* < 0.001, ∆*R*^2^ = 0.293. Elevated *GMD* was associated with lower reported cool detection thresholds, and after adding *GMD* to the model, both sex and PIQ remained significant predictors of cool detection thresholds.

Proportional odds regression models were then fit to the data for children/adolescent, adult, and ASD groups separately to allow for the inclusion of group-specific predictors into the models. The results of the subgroup analyses were very similar to those in the full sample and thus will only be briefly summarized below (see Supplementary Tables S5–S11 for additional information). Of particular note, both best-subset regression models in the child subsample included SP Low Registration scores in the final model, in addition to PIQ. Higher parent-reported low registration (i.e., sensory hyporesponsiveness) on the SP was associated with higher warm thresholds and lower cold thresholds, but these relationships were not statistically significant in either model (*p*s > 0.195). As an additional finding of note, ASD diagnosis was found to be a significant predictor of warm detection threshold in the adult baseline model, adjusted odds ratio (*aOR*) = 2.92, 95% CI [1.07, 7.96], *p* = 0.036. However, this effect was not present after the addition of PIQ in the best-subset regression model, *aOR* = 2.64, 95% CI [0.99, 7.04], *p* = 0.053. Furthermore, after the addition of *GMD* to the model, the effect of ASD diagnosis was attenuated further, *aOR* = 1.71, 95% CI [0.64, 4.61], *p* = 0.287.

## Discussion

Employing a standard method-of-limits psychophysical protocol, we did not find significant differences between individuals with ASD and TD controls in warm or cool detection thresholds. Using equivalence testing procedures, we were able to reject the hypothesis that group differences are present with an effect size of “medium” or larger (|δ| > 0.33). This study addresses limitations of prior work by recruiting larger samples with wide age ranges, accounting for confounding variables in our analyses, and employing robust statistical techniques appropriate for group comparison with highly skewed distributions and outliers. Although our findings conflict with those of Duerden *et al*.^18^, they are consistent with other smaller studies that did not find altered thresholds in individuals with ASD^20,23,24^. However, in concordance with the Duerden study, we did find significant relationships between IQ and thermal detection thresholds, with lower PIQ scores (but not VIQ scores) predicting higher detection thresholds. Notably, we report substantially lower correlations, indicating that the large correlations found by Duerden *et al*. may have been elevated due to their small sample (*n* = 17)^57^.

Proportional-odds regression analyses tended to agree with univariate group comparisons, such that ASD status was not significantly related to thermal detection thresholds, nor were the covariates of sex, age, and counterbalance order (with the exception of the warm detection thresholds in the adult subsample). With regard to significant covariates, only performance IQ was consistently included in every model. The only other predictor to be included in the best-fitting models was caregiver-reported low registration on the sensory profile, a scale that ostensibly measures the behavioral hyporeactivity described in the DSM–5. As would be expected, higher reported hyporeactivity (represented by lower scores on the SP scale) was associated with higher warm and cool detection thresholds, although these effects were not statistically significant.

Once controlling for PIQ, sex also became a significant predictor in all threshold models, with males reporting higher warm detection thresholds and lower cool detection thresholds than females. Although there are challenges in interpretation of significant residualized predictors^58^, this effect of sex is consistent with previous research. Higher sensitivity to thermal stimulation in women was reported in 10 of the 24 psychophysical studies reviewed by Bakkers and colleagues^16^. Additionally, post-hoc comparisons between males and females in our sample (Supplementary Table S1) found males to have significantly higher PIQ scores than females, δ = 0.204, *p* = 0.046, *p*_equiv_ = 0.099. Thus, it is likely that sampling bias introduced a large difference in PIQ between sexes, which confounded the sex effect on thresholds until PIQ was added to the model. Similarly, in the one model where ASD diagnosis emerged as a significant predictor (warm threshold model in adults), the effect was no longer significant after controlling for PIQ. The emergence and disappearance of effects between models with and without PIQ strongly suggests that intelligence can confound meaningful group differences in thermal thresholds as measured by the method of limits. Thus, it is important that future studies on this topic include measures of nonverbal IQ, particularly when evaluating threshold differences between diagnostic groups.

Of note, all best-subset regression models for warm and cool thresholds in this study contained identical predictor sets. This finding is likely driven by the large rank correlation between warm and cool detection thresholds in our sample. While it was historically thought that warm and cool sensations are mediated by separate modality-specific sensory channels^59^, recent animal work suggests innocuous thermal stimuli are sensed by a common set of polymodal nerve fibers^60^. A shared afferent system could explain our strong rank correlations between the two modalities. It is also quite possible that this cross-modal covariance is due to the dependency of both threshold measures on reaction time. Further experiments will be necessary to determine the degree to which warm and cool thresholds covary when measured with both reaction time-dependent and reaction time-independent methods.

After selecting the best predictive models, the *GMD* (i.e., the average absolute difference in temperature between all pairs of trials) was added as an additional predictor representing the intra-individual variability of reported detection thresholds. In all models, *GMD* was a highly significant predictor, providing a large amount of explanatory power to all models (mean ∆*R*^2^ = 0.248, range [0.058, 0.321]). Across the models, increased *GMD* (corresponding to higher intra-individual variability, perhaps due to increased “perceptual noise”) was associated with higher threshold estimates. This result was supported by zero-order correlations between thresholds and corresponding *GMD* values, indicating that the effect of *GMD* on detection threshold was not due to the inclusion of other variables in the regression model. Additionally, analyses of individuals who were outliers in their respective *GMD* distributions suggested that those individuals had substantially higher thresholds than the remainder of the sample. *These results indicate that increased variability between trials systematically biases threshold estimates away from the starting temperature*. As the ASD group in our study exhibited significantly higher *GMD* values than the TD control group, it is quite possible that past reported group differences in thresholds, as well as the small and non-significant group differences in the current study, are a result of differences in measurement precision or within-subject perceptual variability rather than psychophysical threshold *per se*.

Although we have described a robust relationship between variability and detection threshold in this paradigm, it is beyond the scope of this study to determine the underlying cause of this effect. These data, alongside the significant group differences in *GMD*, seem to support the hypothesis that sensory features of ASD are the result of more unreliable perception, as reflected in increased trial-to-trial variability^41–44^. Noisier perceptions in ASD could theoretically delay perceptual decision-making and increase detection thresholds to a small degree^61^. However, it also may be the case that the relationship between threshold and *GMD* is due to the confounding of threshold measurements by participants’ reaction times. Reaction time and its variability have a strong linear relationship^62^, and both of these variables show robust negative correlations with IQ^63–66^. Moreover, individuals with ASD have been found in numerous studies to have significantly larger variabilities in reaction times than controls, with elevated ADHD symptomology often found to mediate this effect^67–70^.

It is well-known that the method of limits algorithm for determining psychophysical thresholds is reaction time–dependent^58^, and several studies have concluded that certain effects of predictor variables on detection thresholds vary depending on whether the method was reaction time–dependent or not^71,72^ (though see also^31,73^). The influence of reaction times on thermal threshold estimates in ASD has yet to be formally investigated and remains a valuable avenue for future work in this area, particularly since all thermal threshold studies in this population to date have utilized reaction time-dependent methods. Additionally, given the known differences in reaction time between ASD and controls, we recommend that future psychophysical investigations in ASD employ reaction time–independent paradigms^74–78^ to avoid potential confounding.

As we were unable to detect meaningful differences between the ASD and TD groups in either warm or cool detection thresholds, these results do not support the hypothesis of thermal hypo-sensitivity in ASD. Thus, this study raises the question of whether clinical observations of apparent indifference to temperature in this population are truly the result of a low-level sensory process. Even in the presence of equivalent perceptual thresholds across groups, there exist myriad ways in which the representation of a suprathreshold stimulus could be altered in ASD compared to controls. To this effect, the most prominent theories of autistic perception (e.g., Excitation-Inhibition imbalance^79^, greater weighting of sensory information in a Bayesian context^80–82^, and higher levels of endogenous neural noise^83^) each include putative explanations for behavioral hypo-reactivity ASD, often without positing increased sensory thresholds (see Ward^61^ for a more complete discussion of these theories). One additional hypothesis based on recent animal work is that sensory hyporeactivity could result from functional alterations in specific neural ensembles that encode the unpleasantness of a given stimulus^84^. It is worth noting that a number of alternative explanations for the sensory features of ASD do not posit differences in low-level sensory processes at all, instead hypothesizing that group differences occur in higher-level neurocognitive processes that modulate the cognitive appraisal of stimuli or subsequent behavioral reactions^85–93^. Given the diverse range of theories that attempt to explain some or all of the sensory features seen in ASD, future research should attempt to distinguish between these competing explanations (e.g., contrasting group differences in low-level sensory processing with group differences in top-down modulation of percepts). By better understanding the neurocognitive underpinnings of sensory features in ASD, this area of research can slowly shift away from descriptive science toward the design of targeted interventions for these often-impairing symptoms.

Strengths of the current study include its comparatively large sample, wide age range of participants, and the inclusion of clinically-relevant covariates. Robust statistical tests were used to compensate for inherently skewed and outlier-prone data, and equivalence testing procedures were able to provide evidence suggesting small to negligible differences in thresholds between diagnostic groups. However, this study also had a number of limitations. As mentioned above, we believe the thresholds estimated in this study may be related to reaction time, raising the possibility of a substantial confound. Without a measure of reaction time to include as a covariate or a reaction time–independent measure of thermal detection threshold, we were unable to assess the magnitude of the confound or determine whether threshold estimates differ between groups after controlling for reaction time. We also were not able to determine whether outliers in the thermal threshold distributions represent participants with truly elevated thresholds, as we would not be able to separate these individuals from those with typical thresholds but slower than average reaction times. If a subgroup with genuinely elevated thermal thresholds is found when using a reaction–time independent task, individuals in this group may possess clinically significant alterations in peripheral neurophysiology, warranting further study. Another limitation is the exclusion of individuals with IQ values in the intellectually disabled range in order to ensure compliance with the experimental tasks. While this subpopulation is often excluded from neuroscientific studies and underrepresented in ASD research at large^94,95^, individuals with low IQ are over-represented in many reports describing hyporeactivity to pain and temperature in ASD^7,8^. Lastly, this investigation was not adequately powered to rule out “small” population effect sizes (i.e., Cliff’s delta value of 0.148, approximately equivalent to a Cohen’s *d* value of 0.2). However, such a small effect would not likely be sufficient to explain the large group differences in behavioral reactivity in ASD.

In conclusion, the findings of the present study appear to support the notion that the behavioral hyporeactivity to thermal stimuli often seen in ASD is not necessarily a reflection of elevated perceptual thresholds. Warm and cool detection thresholds, as measured in a reaction time-dependent manner, were most robustly related to performance IQ, sex, and intra-individual threshold variability. These results support the majority of previous investigations in this area, suggesting that group differences in detection thresholds, if they do exist, are likely not large enough to be clinically meaningful. Further research in this area is thus needed to better understand the sensory and non-sensory processes that contribute to the clinical phenotype of thermosensory hyporeactivity in individuals with ASD.

## Methods

### Participants

#### Adults

Thirty-two adult participants with ASD (21 male, median age 25.50 years) and 24 with typical development (TD) (14 male, median age 29.76 years) were included in the study (Table 1). All included participants were between the ages of 18–54 years and had full-scale IQ scores of at least 70 as measured by the Wechsler Abbreviated Scales of Intelligence—Second Edition (WASI-II)^96^. Full inclusion and exclusion criteria for participants in the study are detailed in the Supplementary Methods. Diagnoses of ASD were confirmed through research-reliable administrations of the Autism Diagnostic Observation Schedule—Second Edition (ADOS-2)^33^ by a licensed clinical psychologist specializing in the assessment of ASD. The definitive judgment of diagnostic status was made based on the clinical judgment of the licensed clinical psychologist, guided but not constrained by ADOS scores. Seven (22%) of the ASD adults were taking medications with psychiatric indications, with the most common being benzodiazepines (*n* = 3).

#### Children/Adolescents

In total, 51 children and adolescents with ASD (41 male, median age 10.03 years) and 35 children and adolescents with TD (26 male, mean age 9.21 years), ages 7–17 years, were included in the study. Inclusion criteria were similar to those for adults, with several exceptions (see Supplementary Methods for more detail). ASD diagnoses were confirmed using clinical judgment, ADOS-2 score, and in a subset of children, the Autism Diagnostic Interview-Revised (ADI-R)^97^. Nineteen of the ASD children (37%) were taking at least one psychiatric medication at the time of participation, with SSRIs (*n* = 9) and psychostimulants (*n* = 10) being the most common.

The current study was conducted in accordance with the Declaration of Helsinki, and all study procedures were approved by the institutional review board of Vanderbilt University. Participants were recruited from the community through fliers and university autism databases. Written informed consent was obtained from all participants 18 years of age or older. Participants under the age of 18 signed written assent forms, and written informed consent was obtained from each minor’s parent or legal guardian. Participants were compensated $20 per hour for their time.

### Measures

Adult participants in both TD and ASD groups completed self-report questionnaires measuring autistic traits (Social Responsiveness Scale–Second Edition: Adult Self-Report^36^) and sensory features (Adolescent/Adult Sensory Profile^38,39^). Primary caregivers of children in both groups completed analogous caregiver-report questionnaires measuring the same constructs (Social Responsiveness Scale–Second Edition: School Age Form^37^ and Sensory Profile^40^, respectively). Individuals of all ages in the ASD group also completed the ADOS-2, and ADOS-2 calibrated severity scores^34–36^ were used as a measure of ASD severity. Brief descriptions of these measures are presented below, and interested readers are directed to the Supplementary Methods for more in-depth reviews of their psychometric properties and usage in the ASD population.

#### Social Responsiveness Scale–Second Edition

The Social Responsiveness Scale–Second Edition (SRS-2)^37^ is a widely-used 65-item measure of quantitative autistic traits in both the general population and individuals with ASD^98^. The form measures autistic traits in children 4–18 years of age via caregiver report or in adults 19 + via self or other report. Items are scored on a 4-point Likert scale, with 0 = *not true*, 1 = *sometimes true*, 2 = *often true*, and 3 = *almost always true*. Total scores on the SRS-2 range from 0–195, with higher scores indicating higher levels of autistic symptomatology. T-scores (M = 50, SD = 10) are also available for individuals based on sex and the specific form used. In the current study, SRS-2 T-Scores based on the total score and form completed were calculated for all participants and used as dimensional measures of autistic traits in further analyses. Additionally, scores on SRS-2 item 42 (Self-report: *I am overly sensitive to certain sounds, textures, or smells*; Caregiver-report: *Seems overly sensitive to certain sounds, textures, or smells*) were additionally included in analyses as a one-item measure of sensory hyperreactivity.

#### Sensory Profile

The Sensory Profile (SP)^40^ is a 125-item caregiver questionnaire that assesses the frequency of a large number of behaviors theoretically related to the child’s sensory experiences. Items are scored on a 5-point Likert scale with *lower* scores indicating higher frequency of abnormal behavior. The questionnaire is based on the conceptual model of Winnie Dunn^11,12^, wherein the combination of sensory threshold (high or low) and behavioral response (passive or active) generates four theoretical sensory quadrants: low registration (low, passive), sensory seeking (low, active), sensory sensitivity (high, passive), and sensory avoiding (high, active). The SP generates scores for each of the four quadrants, as well as modality-specific scores. Caregivers of participants in our child/adolescent group filled out the SP, from which the four quadrant scores were extracted for use in analyses. Of these, only the low Registration, sensory seeking and sensory sensitivity scales were utilized as potential predictors in regression models due to the large (Spearman) correlations between the sensory avoiding subscale and two of the other SP subscales in our sample (sensory sensitivity: *r*_s_ = 0.765; low registration: Spearman’s *r*_s_ = 0.860).

#### Adolescent/Adult Sensory Profile

The Adolescent/Adult Sensory Profile (AASP)^38,39^ is a 60-item self-report questionnaire that assesses a range of attitudes and behaviors theoretically related to sensory processing in individuals 11 years and older. Like the SP, the AASP is organized into subscales based on the four quadrants of Dunn’s theoretical model^11,12^. Items are scored on a 5-point Likert scale from *Almost Never* to *Almost Always*, but unlike the caregiver SP, *higher* scores indicate higher frequency of abnormal behavior. Participants in the adult group completed the AASP, from which the four quadrant scores were extracted for analysis. Of these, only the low registration, sensory seeking and sensory sensitivity scales were utilized as potential predictors in regression models due to the large (Spearman) correlation between the sensory sensitivity and sensory avoiding subscales in our sample (*r*_s_ = 0.831).

#### ADOS-2

The Autism Diagnostic Observation Schedule–Second Edition (ADOS-2)^34,99,100^ is a structured clinician-administered assessment of autism features typically used to establish a diagnosis of ASD. Multiple modules are available for use with individuals of different ages and verbal abilities. Scores from ADOS-2 items are combined to form a total score, as well as subscale scores for the items reflecting the two DSM-congruent ASD domains of social affect (SA) and restricted/repetitive behaviors (RRB). Calibrated severity scores (CSS)^35,36,100,101^ are also available, which allow ADOS-2 total and subscale scores from different modules to be compared on a common 1–10 metric that is minimally related to age and IQ. ASD participants in our sample were administered the ADOS-2 module 3 or 4, based on age and developmental level, by a licensed clinical psychologist trained to research reliability on the measure. Raw total scores were extracted and converted to overall CSS, which were then used as measures of ASD severity in further analyses. Because of recent findings questioning the reliability of ADOS-2 RRB scores^102^, we chose not to utilize the separate SA and RRB CSS as predictors in our regression models.

### Thermal detection task

The thermal detection task took place in a dedicated sensory testing room within Vanderbilt Psychiatric Hospital, which was maintained at a constant temperature. Thermal stimuli were delivered using a Peltier device with a 30 mm × 30 mm thermoconducting surface (TSA-II – NeuroSensory Analyzer, Medoc, Israel), which was attached to the right thenar palm of each participant using a Velcro strap. The thermode was set to a baseline temperature of 32 °C, approximately the resting temperature of the skin. While in contact with the thermode, participants completed alternating blocks of trials assessing warm detection and cool detection thresholds. Using a modified Marstock method-of-limits protocol^103^, the temperature was increased or decreased at a rate of 1 °C/s until the participant indicated a sensation of warmth or cold via mouse click. Upper and lower temperature boundaries were set at 50° and 0 °C, respectively, to preclude any possibility of tissue damage. When the participant indicated a warm or cool sensation, the thermode temperature was captured and recorded using PC-based software, subsequently returning to baseline at a rate of 3.5 °C/s. The stimuli were applied in alternating blocks of five warm or five cool trials, with the block order counterbalanced across participants. Participants completed two blocks of each trial type for a total of 10 warm and 10 cool trials.

Warm and cool detection threshold values were quantified as the change in temperature from the baseline of 32 °C required for a participant to indicate a sensation of warmth or cold. Threshold estimates for each participant were obtained by calculating the medians of the 10 trials using the Harrell-Davis quantile estimator^104,105^, which performs better than the traditional median estimator in small samples and skewed distributions^106^. Pooling of trials across blocks was supported by excellent intraclass correlations between Harrell-Davis median values derived from each block of trials, warm trials: *ICC*(3,2) = 0.92, 95% CI [0.88, 0.94], cool trials: *ICC*(3,2) = 0.93, 95% CI [0.89, 0.95]. We also assessed intra-individual variation in threshold temperature across trials by calculating the Gini’s mean difference (*GMD*)^42–44^, a robust and highly efficient measure of relative dispersion equal to the mean of absolute differences between all pairs of values in the set. Higher *GMD* values indicate more variable responses across the thermal detection trials, reflecting lower precision of the single-subject threshold estimates, a hypothesized correlate of increased perceptual noise^41^. The functions *hdquantile* and *GiniMd* in the *Hmisc* R package^107^ were used to compute the Harrell-Davis quantile and *GMD* values in our analyses.

### Data analysis

#### Group Comparisons

Demographics, warm and cool detection thresholds, *GMD*s for each trial type, and scores on self-report measures were compared between the ASD and TD groups, with additional ASD-TD comparisons utilizing only the adult and child/adolescent subsamples. Categorical variables were compared between groups using the Pearson chi-square test without continuity correction. As the majority of continuous variables violated the assumptions of *t*-tests, these variables were compared using Cliff’s delta^45–47,50,108^, a robust, non-parametric effect-size statistic that can be used to test differences in distributions between groups. Delta estimates the probability that a randomly selected observation from one group is larger than a randomly selected observation from another group, minus the reverse probability. Values of δ range from −1 to 1, with a value of 0 indicating complete overlap of groups and values of −1 or 1 indicating all values in one group being larger than all values in the other. Under conditions of normality and homoskedasticity, Cliff’s delta can be equated to Cohen’s *d*, with δ values of 0.148, 0.33, and 0.474 corresponding to the oft-cited small, medium, and large Cohen’s *d* benchmarks of 0.2, 0.5, and 0.8^109^.

In addition to standard null hypothesis significance testing with δ, equivalence testing was also conducted using the two one-sided tests (TOST) procedure^48,49,110^ using one-tailed Cliff’s delta. A significant *p* value in the equivalence test allows us to draw the conclusion of statistical equivalence (i.e., the difference between groups is smaller than the smallest effect size of interest, and thus groups do not meaningfully differ). The smallest effect-size of interest was set to δ = ±0.33, because (a) this value constituted the boundary for a “medium” effect size^109^ and (b) a Monte Carlo power analysis (*B* = 10,000 samples) using a population δ of 0, the sample sizes of the ASD and TD groups (83 and 59, respectively), and homoskedastic normally distributed variables calculated substantial power (0.926) to detect equivalency at the 0.05 level. Although we chose to use these same bounds for equivalence tests in the child/adolescent and adult subsamples, the power to detect equivalence at these smaller sample sizes was substantially lower (0.378 and 0.689 for adults and children/adolescents, respectively). All analyses were performed in the R statistical computing environment, with the *orddom* package^111^ used to compute Cliff’s delta.

#### Correlation Analyses

Zero-order correlations between psychophysical, demographic, and behavioral variables were examined using Spearman rank correlations (see Supplementary Tables 2–4 for full correlation matrices). Correlation significance was tested with a Z-transformation using the standard error estimate proposed by Caruso & Cliff^112,113^. Equivalence tests (based on the TOST procedure) were also conducted using one-tailed Z-tests^114^ and equivalence bounds of *r*_s_ =  ± 0.30 (a “medium” effect according to Cohen^109^). Power to detect equivalence at the *r*_s_ =  ± 0.30 (assessed by Monte Carlo power analysis with *B* = 10,000 samples from an uncorrelated bivariate normal population) was 0.951 at *N* = 142 (equivalent to the full sample size), but substantially lower for analyses in subgroups (0.470, 0.752, and 0.732 for the *n*s of the adult, child/adolescent, and ASD subsamples). Comparisons between dependent and independent correlations were tested using the confidence interval methods proposed by Zou^115^, implemented in the *cocor* R package^116^.

#### Regression Models

In order to determine the effects of various predictor variables on thermal thresholds while controlling for covariates, we conducted a multiple regression analysis. However, because the thermal threshold variables were heavily skewed and multiple linear regression assumptions were violated, we chose to conduct a proportional-odds logistic regression using the cumulative probability model (CPM)^51,52^, which is appropriate for use with continuous outcomes. The CPM is a semi-parametric regression model that functions as a multi-predictor generalization of the Wilcoxon–Mann–Whitney test. Additional details on the CPM can be found in the Supplementary Methods.

Regression models were fit in three steps. Initially, a baseline model was fit, in which thermal detection threshold was regressed on diagnostic group (ASD vs. TD), age (in years), sex, and counterbalance order (warm block first vs. cool block first). Additional predictors (verbal IQ, performance IQ, SRS T-score, SRS sensory item score) were added in a second step by best-subset regression with the Bayesian Information Criterion (BIC)^117,118^. Additionally, BIC weights^119^ were used to quantify the probability that the chosen model was the best model, the superiority of the best-fitting model over the closest competitor and baseline models, and the probability that each predictor is included in the best model. In the third step, the corresponding warm or cool *GMD*, which we hypothesized to be strongly predictive of the detection threshold, was added to the regression model, allowing us to test which predictors remained significant after accounting for individual differences in measurement precision. Due to the presence of several group-specific predictor variables (e.g., SP scales for children, AASP scales for adults, ADOS-2 CSS and medication status for the ASD group), regression models were fit on three specific subsamples (children/adolescents only, adults only, ASD only) as well as the combined sample. All statistical analyses were conducted in R, with the *rms* package^120^ used to fit the CPMs. Missing values were handled with 20-fold multiple imputation using the *Hmisc* package^103,121^.

## Supplementary information

Supplementary Information

## Data Availability

The datasets used and/or analyzed during the current study are available from the corresponding author on reasonable request.
