# Supplementary material for: Thermal Perceptual Thresholds are typical in Autism Spectrum Disorder but Strongly Related to Intra-individual Response Variability
Source: Sci Rep. 2019 Aug 29;9:12595. doi: 10.1038/s41598-019-49103-2 (PMC6715703; doi:10.1038/s41598-019-49103-2)
Supplement: Supplementary file 1 — Supplementary Information [file 41598_2019_49103_MOESM1_ESM.pdf]

Supplementary Information for "Thermal Perceptual Thresholds are typical in Autism Spectrum Disorder but Strongly Related to Intra-individual Response Variability"

Zachary J Williams*, Michelle D. Failla, Samona L. Davis, Brynna H. Heflin, Christian D. Okitondo, David J. Moore, & Carissa J. Cascio

Supplementary Table S1. Descriptive statistics and male-female comparisons

Variable	N (F/M)	F [*Mdn*, (Q_1, Q_3)]	M [*Mdn*, (Q_1, Q_3)]	δ (90% CI)	P_{H0}	P_{equiv}
Age (Years)	40/102	19.81 (9.19, 30.46)	13.21 (9.09, 22.48)	-0.153 (-0.34, 0.046)	0.199	0.060
Verbal IQ	39/99	105.88 (90.98, 121.57)	103.8 (96.46, 112.88)	-0.044 (-0.244, 0.159)	0.724	**0.008**
Performance IQ	39/99	103.03 (95.52, 112.88)	109.39 (96.98, 122.62)	0.204 (0.033, 0.363)	**0.046**	0.099
Full-scale IQ	39/99	106.69 (93.73, 115.63)	107.53 (97.02, 117.95)	0.074 (-0.112, 0.255)	0.514	**0.009**
SRS-2 Total T-score	29/82	59.84 (44.07, 77.51)	63.3 (48.07, 73.85)	0.022 (-0.199, 0.241)	0.870	**0.009**
SRS-2 Item 42 (0–3)	31/88	1.00 (0.01, 2.56)	1.03 (0.11, 2.32)	0.05 (-0.156, 0.252)	0.692	**0.010**
Warm Threshold (°C)	40/102	1.48 (1.00, 2.34)	1.89 (1.25, 2.73)	0.219 (0.035, 0.389)	**0.045**	0.146
Cool Threshold (°C)	40/102	2.13 (1.63, 2.62)	2.37 (1.83, 3.24)	0.203 (0.021, 0.372)	0.061	0.111
Warm *GMD* (°C)	40/102	0.44 (0.27, 0.75)	0.58 (0.36, 0.93)	0.200 (0.019, 0.367)	0.063	0.104
Cool *GMD* (°C)	40/102	0.43 (0.21, 0.71)	0.51 (0.30, 0.96)	0.180 (-0.007, 0.354)	0.107	0.080

Note. Threshold values indicate changes (in °C) from the baseline temperature of 32°C. δ = Cliff's (1993) delta statistic (an effect size metric); Q_1 = first quartile; Q_3 = third quartile; P_{H0} = *p*-value for test of null hypothesis of no effect; P_{equiv} = *p*-value for equivalence test (H_0: $|\delta| \geq 0.33$, H_A: $|\delta| < 0.33$); AASP = Adolescent/Adult Sensory Profile; *GMD* = Gini's Mean Difference; SP = Sensory Profile; SRS-2 = Social Responsiveness Scale – Second Edition; SRS-2 item 42 = "*I am overly sensitive to certain sounds, textures, or smells*" (self-report) or "*Seems overly sensitive to certain sounds, textures, or smells*" (caregiver report).

Table S2a. Spearman correlations (below diagonal) and 90% confidence intervals (above diagonal) – Entire sample

	**WDT**	**CDT**	**GMD (warm)**	**GMD (cool)**	**Age (Years)**	**VIQ**	**PIQ**	**FSIQ**	**SRS T-score**	**SRS item 42**
**WDT**	—	(0.79, 0.88)	(0.564, 0.732)	(0.516, 0.699)	**(-0.217, 0.06)**	**(-0.178, 0.103)**	(-0.367, -0.097)	**(-0.287, -0.01)**	**(-0.041, 0.27)**	**(-0.109, 0.193)**
**CDT**	**0.840**	—	(0.587, 0.748)	(0.623, 0.773)	(-0.309, -0.037)	**(-0.247, 0.032)**	(-0.445, -0.187)	(-0.371, -0.102)	**(-0.063, 0.249)**	**(-0.075, 0.227)**
**GMD (warm)**	**0.656**	**0.676**	—	(0.501, 0.688)	(-0.494, -0.25)	**(-0.269, 0.009)**	(-0.357, -0.086)	**(-0.327, -0.053)**	(0.083, 0.383)	**(-0.09, 0.212)**
**GMD (cool)**	**0.616**	**0.706**	**0.603**	—	(-0.431, -0.174)	**(-0.242, 0.038)**	(-0.433, -0.173)	(-0.364, -0.094)	(0.005, 0.313)	**(-0.118, 0.184)**
**Age (Years)**	-0.080	**-0.176**	**-0.378**	**-0.308**	—	**(-0.244, 0.035)**	**(-0.161, 0.119)**	**(-0.225, 0.055)**	**(-0.246, 0.066)**	(0.006, 0.304)
**VIQ**	-0.038	-0.110	-0.133	-0.104	-0.107	—	(0.376, 0.598)	(0.818, 0.898)	(-0.36, -0.052)	(-0.335, -0.037)
**PIQ**	**-0.237**	**-0.322**	**-0.226**	**-0.308**	-0.021	**0.495**	—	(0.798, 0.885)	**(-0.192, 0.126)**	**(-0.227, 0.077)**
**FSIQ**	-0.151	**-0.241**	**-0.193**	**-0.234**	-0.086	**0.863**	**0.847**	—	**(-0.3, 0.014)**	(-0.304, -0.003)
**SRS T-score**	0.117	0.096	**0.239**	0.163	-0.093	**-0.211**	-0.034	-0.147	—	(0.559, 0.749)
**SRS item 42**	0.043	0.078	0.063	0.034	0.158	**-0.190**	-0.077	-0.157	**0.664**	—

Note. Bolded values indicate $p < 0.05$. SRS item 42 = "*I am overly sensitive to certain sounds, textures, or smells*" (self-report) or "*Seems overly sensitive to certain sounds, textures, or smells*" (caregiver report).

Table S2b. P-values for null-hypothesis significance tests (below diagonal) and $|r_s| \geq 0.3$ equivalence tests (above diagonal)

	**WDT**	**CDT**	**GMD (warm)**	**GMD (cool)**	**Age (Years)**	**VIQ**	**PIQ**	**FSIQ**	**SRS T-score**	**SRS item 42**
**WDT**	—	> 0.999	> 0.999	> 0.999	**0.004**	**0.001**	0.217	**0.035**	**0.024**	**0.002**
**CDT**	**< 0.001**	—	> 0.999	> 0.999	0.062	**0.011**	0.611	0.233	**0.014**	**0.006**
**GMD (warm)**	**< 0.001**	**< 0.001**	—	> 0.999	0.880	**0.006**	0.116	**0.044**	0.196	**0.002**
**GMD (cool)**	**< 0.001**	**< 0.001**	**< 0.001**	—	0.628	**0.012**	0.489	0.215	0.053	**0.001**
**Age (Years)**	0.345	**0.038**	**< 0.001**	**< 0.001**	—	**0.010**	**< 0.001**	**0.005**	**0.012**	0.055
**VIQ**	0.655	0.203	0.124	0.227	0.215	—	0.995	> 0.999	0.167	0.109
**PIQ**	**0.006**	**< 0.001**	**0.008**	**< 0.001**	0.804	**< 0.001**	—	> 0.999	**0.002**	**0.007**
**FSIQ**	0.078	**0.005**	**0.024**	**0.006**	0.316	**< 0.001**	**< 0.001**	—	0.050	0.055
**SRS T-score**	0.223	0.319	**0.013**	0.090	0.335	**0.030**	0.728	0.133	—	> 0.999
**SRS item 42**	0.644	0.401	0.499	0.717	0.088	**0.042**	0.413	0.093	**< 0.001**	—

Note. Bolded values indicate $p < 0.05$. SRS item 42 = "*I am overly sensitive to certain sounds, textures, or smells*" (self-report) or "*Seems overly sensitive to certain sounds, textures, or smells*" (caregiver report).

Table S3a. Spearman correlations (below diagonal) and 90% confidence intervals (above diagonal) – Adults only

	WDT	CDT	GMD (warm)	GMD (cool)	Age (Years)	VIQ	PIQ	FSIQ	SRS T-score	Low Registration	Sensory Seeking	Sensory Sensitivity	Sensory Avoiding	SRS Item 42
**WDT**	—	(0.779 0.911)	(0.595, 0.822)	(0.536, 0.79)	**(-0.163, 0.278)**	(-0.452, -0.019)	(-0.601, -0.216)	(-0.539, -0.129)	(-0.105, 0.386)	**(-0.218, 0.279)**	**(-0.235, 0.262)**	(-0.107, 0.385)	**(-0.211, 0.287)**	(-0.09, 0.4)
**CDT**	**0.858**	—	(0.561, 0.804)	(0.488, 0.764)	(-0.082, 0.355)	(-0.394, 0.05)	(-0.624, -0.249)	(-0.525, -0.111)	**(-0.200, 0.298)**	**(-0.275, 0.222)**	(-0.126, 0.368)	(-0.151, 0.345)	**(-0.283, 0.215)**	(-0.026, 0.454)
**GMD (warm)**	**0.728**	**0.702**	—	(0.347, 0.68)	(-0.07, 0.365)	(-0.42, 0.02)	(-0.492, -0.068)	(-0.464, -0.032)	(0.063, 0.525)	(-0.141, 0.354)	(-0.457, 0.023)	(-0.034, 0.447)	(-0.128, 0.366)	(-0.026, 0.454)
**GMD (cool)**	**0.683**	**0.646**	**0.534**	—	**(-0.163, 0.278)**	(-0.427, 0.012)	(-0.451, -0.017)	(-0.456, -0.023)	(-0.055, 0.43)	(-0.442, 0.04)	**(-0.206, 0.292)**	(-0.373, 0.12)	(-0.423, 0.063)	(-0.038, 0.444)
**Age (Years)**	**0.061**	0.144	0.155	0.061	—	(-0.03, 0.412)	(-0.138, 0.314)	(-0.079, 0.369)	(-0.327, 0.169)	(-0.568, -0.123)	(-0.044, 0.439)	(-0.33, 0.167)	(-0.407, 0.082)	**(-0.232, 0.265)**
**VIQ**	-0.248	-0.181	-0.211	-0.219	0.201	—	(0.414, 0.728)	(0.837, 0.938)	(-0.485, 0)	(-0.557, -0.095)	(-0.032, 0.459)	(-0.524, -0.05)	(-0.411, 0.09)	(-0.404, 0.092)
**PIQ**	**-0.428**	**-0.457**	**-0.295**	-0.247	0.093	**0.593**	—	(0.789, 0.917)	**(-0.231, 0.278)**	(-0.401, 0.101)	(-0.406, 0.096)	**(-0.297, 0.212)**	**(-0.222, 0.287)**	(-0.376, 0.123)
**FSIQ**	**-0.351**	**-0.334**	-0.261	-0.252	0.153	**0.898**	**0.867**	—	(-0.343, 0.164)	(-0.483, 0.003)	**(-0.236, 0.274)**	(-0.396, 0.107)	**(-0.289, 0.221)**	(-0.404, 0.092)
**SRS T-score**	0.15	0.052	0.312	0.199	-0.084	-0.259	0.025	-0.096	—	(0.505, 0.816)	(-0.687, -0.258)	(0.437, 0.783)	(0.502, 0.815)	(0.443, 0.771)
**Low Registration**	0.032	-0.028	0.113	-0.214	**-0.366**	**-0.347**	-0.161	-0.256	**0.692**	—	(-0.623, -0.202)	(0.673, 0.877)	(0.64, 0.862)	(0.346, 0.736)
**Sensory Seeking**	0.014	0.129	-0.231	0.046	0.211	0.228	-0.166	0.02	**-0.503**	**-0.436**	—	(-0.618, -0.195)	(-0.629, -0.212)	(-0.431, 0.101)
**Sensory Sensitivity**	0.148	0.103	0.22	-0.135	-0.087	-0.306	-0.046	-0.154	**0.642**	**0.796**	**-0.429**	—	(0.782, 0.922)	(0.357, 0.742)
**Sensory Avoiding**	0.04	-0.036	0.126	-0.191	-0.173	-0.172	0.035	-0.036	**0.689**	**0.774**	**-0.445**	**0.868**	—	(0.259, 0.687)
**SRS Item 42**	0.165	0.228	0.228	0.216	0.018	-0.167	-0.135	-0.166	**0.635**	**0.573**	-0.178	**0.581**	**0.504**	—

Note. Sensory quadrant scores (Low Registration, Sensory Seeking, Sensory Sensitivity, Sensory Avoiding) are based on AASP scales. Bolded values indicate $p < 0.05$. SRS item 42 = "*I am overly sensitive to certain sounds, textures, or smells*" (self-report).

Table S3b. P-values for null-hypothesis significance tests (below diagonal) and $|r_s| \geq 0.3$ equivalence tests (above diagonal) – Adults only

	WDT	CDT	GMD (warm)	GMD (cool)	Age (Years)	VIQ	PIQ	FSIQ	SRS T-score	Low Registration	Sensory Seeking	Sensory Sensitivity	Sensory Avoiding	SRS Item 42
**WDT**	—	> 0.999	> 0.999	0.999	**0.034**	0.346	0.847	0.654	0.155	**0.037**	**0.028**	0.152	**0.041**	0.180
**CDT**	**< 0.001**	—	> 0.999	0.999	0.115	0.187	0.897	0.605	**0.048**	**0.034**	0.124	0.093	**0.039**	0.310
**GMD (warm)**	**< 0.001**	**< 0.001**	—	0.978	0.132	0.252	0.484	0.383	0.534	0.104	0.317	0.291	0.121	0.310
**GMD (cool)**	**< 0.001**	**< 0.001**	**< 0.001**	—	**0.034**	0.270	0.343	0.358	0.246	0.278	**0.044**	0.132	0.230	0.284
**Age (Years)**	0.657	0.293	0.282	0.436	—	0.229	0.062	0.136	0.074	0.681	0.271	0.076	0.194	**0.030**
**VIQ**	0.076	0.197	0.256	0.171	0.151	—	0.994	> 0.999	0.391	0.627	0.315	0.516	0.197	0.185
**PIQ**	**0.002**	**0.001**	**0.045**	0.103	0.510	**< 0.001**	—	> 0.999	**0.036**	0.178	0.187	**0.048**	**0.042**	0.135
**FSIQ**	**0.011**	**0.015**	0.105	0.092	0.277	**< 0.001**	**< 0.001**	—	0.090	0.385	**0.034**	0.168	**0.042**	0.185
**SRS T-score**	0.333	0.735	0.081	0.346	0.586	0.100	0.874	0.546	—	0.999	0.917	0.994	0.999	0.996
**Low Registration**	0.834	0.855	0.513	0.104	**0.015**	**0.026**	0.311	0.103	**< 0.001**	—	0.838	> 0.999	> 0.999	0.974
**Sensory Seeking**	0.926	0.406	0.127	0.552	0.172	0.149	0.296	0.898	**0.002**	**0.003**	—	0.826	0.855	0.225
**Sensory Sensitivity**	0.339	0.505	0.282	0.215	0.574	0.051	0.773	0.330	**< 0.001**	**< 0.001**	**0.004**	—	> 0.999	0.977
**Sensory Avoiding**	0.793	0.814	0.551	0.110	0.263	0.279	0.825	0.818	**< 0.001**	**< 0.001**	**0.003**	**< 0.001**	—	0.918
**SRS Item 42**	0.287	0.140	0.179	0.172	0.908	0.288	0.390	0.288	**< 0.001**	**< 0.001**	0.293	**< 0.001**	**0.002**	—

Note. Sensory quadrant scores (Low Registration, Sensory Seeking, Sensory Sensitivity, Sensory Avoiding) are based on AASP scales. Bolded values indicate $p < 0.05$. SRS item 42 = "*I am overly sensitive to certain sounds, textures, or smells*" (self-report).

Table S4a. Spearman correlations (below diagonal) and 90% confidence intervals (above diagonal) – Children/adolescents only

	**WDT**	**CDT**	**GMD (warm)**	**GMD (cool)**	**Age (Years)**	**VIQ**	**PIQ**	**FSIQ**	**SRS T-score**	**Low Registration**	**Sensory Seeking**	**Sensory Sensitivity**	**Sensory Avoiding**	**SP Item 42**	**SRS Item 42**
**WDT**	—	(0.754, 0.879)	(0.478, 0.715)	(0.453, 0.699)	**(-0.238, 0.118)**	**(-0.129, 0.229)**	(-0.33, 0.022)	**(-0.231, 0.127)**	(-0.09, 0.31)	**(-0.224, 0.159)**	**(-0.204, 0.179)**	**(-0.286, 0.096)**	**(-0.224, 0.159)**	**(-0.096, 0.286)**	**(-0.197, 0.183)**
**CDT**	**0.827**	—	(0.454, 0.700)	(0.651, 0.822)	(-0.322, 0.030)	**(-0.262, 0.094)**	(-0.437, -0.100)	(-0.380, -0.033)	**(-0.107, 0.295)**	**(-0.280, 0.103)**	**(-0.257, 0.126)**	**(-0.279, 0.103)**	**(-0.223, 0.161)**	**(-0.124, 0.259)**	**(-0.187, 0.193)**
**GMD (warm)**	**0.610**	**0.590**	—	(0.444, 0.693)	(-0.391, -0.048)	**(-0.266, 0.09)**	(-0.423, -0.083)	(-0.363, -0.014)	(0.014, 0.404)	**(-0.291, 0.091)**	**(-0.308, 0.073)**	**(-0.295, 0.086)**	(-0.345, 0.032)	**(-0.269, 0.114)**	**(-0.125, 0.256)**
**GMD (cool)**	**0.589**	**0.748**	**0.582**	—	(-0.407, -0.067)	**(-0.252, 0.105)**	(-0.538, -0.226)	(-0.443, -0.106)	(-0.079, 0.321)	**(-0.245, 0.139)**	**(-0.194, 0.189)**	(-0.365, 0.01)	**(-0.298, 0.083)**	**(-0.133, 0.251)**	**(-0.183, 0.198)**
**Age (Years)**	-0.062	-0.151	**-0.227**	**-0.245**	—	**(-0.424, -0.084)**	**(-0.166, 0.191)**	(-0.317, 0.037)	**(-0.154, 0.249)**	**(-0.292, 0.09)**	(-0.036, 0.342)	**(-0.214, 0.169)**	**(-0.21, 0.173)**	**(-0.268, 0.115)**	(-0.075, 0.303)
**VIQ**	0.051	-0.087	-0.091	-0.076	**-0.262**	—	(0.264, 0.567)	(0.78, 0.894)	(-0.403, -0.009)	(0.049, 0.419)	(-0.068, 0.315)	(0.064, 0.432)	(0.031, 0.404)	(-0.004, 0.373)	(-0.387, -0.014)
**PIQ**	-0.159	**-0.277**	**-0.261**	**-0.393**	0.013	**0.427**	—	(0.749, 0.877)	**(-0.293, 0.112)**	**(-0.121, 0.264)**	**(-0.2, 0.186)**	(-0.04, 0.341)	(-0.031, 0.349)	**(-0.113, 0.272)**	**(-0.23, 0.154)**
**FSIQ**	-0.054	-0.213	-0.194	**-0.283**	-0.145	**0.846**	**0.823**	—	(-0.387, 0.009)	(-0.01, 0.368)	**(-0.118, 0.267)**	(0.062, 0.43)	(0.041, 0.413)	(-0.036, 0.344)	(-0.331, 0.048)
**SRS T-score**	0.115	0.098	0.218	0.126	0.049	-0.214	-0.094	-0.197	—	(-0.866, -0.691)	(-0.719, -0.424)	(-0.785, -0.537)	(-0.878, -0.716)	(-0.686, -0.37)	(0.619, 0.823)
**Low Registration**	-0.034	-0.092	-0.104	-0.055	-0.105	**0.243**	0.074	0.186	**-0.794**	—	(0.467, 0.723)	(0.617, 0.813)	(0.793, 0.906)	(0.526, 0.759)	(-0.733, -0.47)
**Sensory Seeking**	-0.013	-0.068	-0.122	-0.003	0.159	0.128	-0.007	0.078	**-0.591**	**0.611**	—	(0.472, 0.727)	(0.459, 0.718)	(0.418, 0.692)	(-0.577, -0.235)
**Sensory Sensitivity**	-0.099	-0.092	-0.109	-0.184	-0.023	**0.257**	0.156	**0.256**	**-0.680**	**0.729**	**0.615**	—	(0.665, 0.839)	(0.436, 0.704)	(-0.678, -0.382)
**Sensory Avoiding**	-0.034	-0.032	-0.162	-0.112	-0.019	0.226	0.165	**0.235**	**-0.812**	**0.860**	**0.604**	**0.765**	—	(0.492, 0.739)	(-0.746, -0.49)
**SP Item 42**	0.099	0.070	-0.080	0.061	-0.079	0.192	0.083	0.160	**-0.547**	**0.658**	**0.571**	**0.586**	**0.631**	—	(-0.602, -0.271)
**SRS Item 42**	-0.007	0.003	0.068	0.008	0.118	-0.208	-0.039	-0.147	**0.737**	**-0.619**	**-0.420**	**-0.547**	**-0.635**	**-0.452**	—

Note. Sensory quadrant scores (Low Registration, Sensory Seeking, Sensory Sensitivity, Sensory Avoiding) are based on SP caregiver questionnaire scales. Bolded values indicate $p < 0.05$. SP item 42 = "*Decreased awareness of pain and temperature*" (caregiver report). SRS item 42 = "*Seems overly sensitive to certain sounds, textures, or smells*" (caregiver report).

Table S4b. P-values for null-hypothesis significance tests (below diagonal) and $|r_s| \geq 0.3$ *equivalence tests (above diagonal) – Children/adolescents only*

	**WDT**	**CDT**	**GMD (warm)**	**GMD (cool)**	**Age (Years)**	**VIQ**	**PIQ**	**FSIQ**	**SRS T-score**	**Low Registration**	**Sensory Seeking**	**Sensory Sensitivity**	**Sensory Avoiding**	**SP Item 42**	**SRS Item 42**
**WDT**	—	> 0.999	> 0.999	0.999	**0.012**	**0.010**	0.090	**0.010**	0.060	**0.010**	**0.006**	**0.038**	**0.010**	**0.038**	**0.005**
**CDT**	**< 0.001**	—	0.999	> 0.999	0.077	**0.022**	0.412	0.203	**0.045**	**0.034**	**0.021**	**0.033**	**0.009**	**0.022**	**0.004**
**GMD (warm)**	**< 0.001**	**< 0.001**	NA	0.999	0.239	**0.024**	0.353	0.156	0.243	**0.042**	**0.058**	**0.046**	0.111	**0.027**	**0.020**
**GMD (cool)**	**< 0.001**	**< 0.001**	**< 0.001**	NA	0.295	**0.017**	0.826	0.434	0.073	**0.016**	**0.005**	0.151	**0.048**	**0.018**	**0.005**
**Age (Years)**	0.571	0.169	**0.038**	**0.025**	—	0.358	**0.003**	0.070	**0.018**	**0.043**	0.105	**0.008**	**0.007**	**0.026**	0.053
**VIQ**	0.641	0.431	0.410	0.492	**0.016**	—	0.902	> 0.999	0.235	0.304	0.066	0.351	0.254	0.170	0.206
**PIQ**	0.149	**0.011**	**0.017**	**< 0.001**	0.907	**< 0.001**	—	> 0.999	**0.044**	**0.024**	**0.005**	0.102	0.118	**0.029**	**0.011**
**FSIQ**	0.626	0.052	0.077	**0.010**	0.189	**< 0.001**	**< 0.001**	—	0.193	0.157	**0.026**	0.345	0.282	0.108	0.088
**SRS T-score**	0.357	0.432	0.079	0.310	0.693	0.086	0.453	0.116	—	> 0.999	0.996	> 0.999	> 0.999	0.987	> 0.999
**Low Registration**	0.776	0.437	0.380	0.643	0.375	**0.040**	0.533	0.118	**< 0.001**	—	0.999	> 0.999	> 0.999	> 0.999	0.999
**Sensory Seeking**	0.914	0.566	0.304	0.982	0.180	0.282	0.952	0.515	**< 0.001**	**< 0.001**	—	0.999	0.999	0.997	0.862
**Sensory Sensitivity**	0.402	0.439	0.359	0.119	0.844	**0.030**	0.191	**0.031**	**< 0.001**	**< 0.001**	**< 0.001**	—	> 0.999	0.998	0.991
**Sensory Avoiding**	0.776	0.786	0.170	0.346	0.870	0.057	0.165	**0.047**	**< 0.001**	**< 0.001**	**< 0.001**	**< 0.001**	—	> 0.999	> 0.999
**SP Item 42**	0.404	0.556	0.499	0.604	0.502	0.106	0.487	0.180	**< 0.001**	**< 0.001**	**< 0.001**	**< 0.001**	**< 0.001**	—	0.918
**SRS Item 42**	0.950	0.979	0.562	0.945	0.314	0.078	0.739	0.213	**< 0.001**	**< 0.001**	**< 0.001**	**< 0.001**	**< 0.001**	**< 0.001**	—

Note. Sensory quadrant scores (Low Registration, Sensory Seeking, Sensory Sensitivity, Sensory Avoiding) are based on SP caregiver questionnaire scales. Bolded values indicate $p < 0.05$. SP item 42 = "*Decreased awareness of pain and temperature*" (caregiver report). SRS item 42 = "*Seems overly sensitive to certain sounds, textures, or smells*" (caregiver report).

Supplementary Table S5. Regression models for warm and cool detection thresholds – Adults only

Warm Step 1: Baseline Model

Predictor	aOR (95% CI)	Wald χ^2	P
**Diagnosis (ASD)**	**2.92 (1.07, 7.96)**	**4.41**	**0.036***
Sex (Male)	1.92 (0.71, 5.16)	1.66	0.198
Age (Years)	1.03 (0.97, 1.09)	1.06	0.303
Counterbalance	1.04 (0.40, 2.72)	0.01	0.932
**Model Fit**	$\chi^2(4) = 7.24$	$p = 0.124$	$R^2 = 0.121$

Cool Step 1: Baseline Model

Predictor	aOR (95% CI)	Wald χ^2	P
Diagnosis (ASD)	1.94 (0.75 5.04)	1.86	0.172
Sex (Male)	2.06 (0.77, 5.48)	2.08	0.149
Age (Years)	1.05 (0.98, 1.11)	1.90	0.168
Counterbalance	1.34 (0.52, 3.44)	0.36	0.546
**Model Fit**	$\chi^2(4) = 5.61$	$p = 0.230$	$R^2 = 0.095$

Warm Step 2: Best-subset Regression Model

Predictor	aOR (95% CI)	Wald χ^2	P
Diagnosis (ASD)	2.64 (0.99, 7.04)	3.73	0.053
**Sex (Male)**	**3.21 (1.16, 8.87)**	**5.06**	**0.025***
**Age (Years)**	**1.07 (1.01, 1.14)**	**5.02**	**0.025***
Counterbalance	2.05 (0.74, 5.68)	1.93	0.165
**PIQ**	**0.94 (0.91, 0.97)**	**15.01**	**< 0.001***
**Model Fit**	$\chi^2(5) = 23.13$	$p < 0.001$*	$R^2 = 0.338$

Cool Step 2: Best-subset Regression Model

Predictor	aOR (95% CI)	Wald χ^2	P
Diagnosis (ASD)	1.82 (0.69, 4.78)	1.48	0.224
**Sex (Male)**	**2.88 (1.05, 7.88)**	**4.23**	**0.040***
**Age (Years)**	**1.09 (1.02, 1.17)**	**6.10**	**0.014***
Counterbalance	2.23 (0.83, 6.01)	2.50	0.114
**PIQ**	**0.94 (0.91, 0.97)**	**16.79**	**<0.001***
**Model Fit**	$\chi^2(5) = 23.05$	$p < 0.001$*	$R^2 = 0.338$

Warm Step 3: Best-subset Model with GMD

Predictor	aOR (95% CI)	Wald χ^2	P
Diagnosis (ASD)	1.71 (0.64, 4.61)	1.13	0.287
**Sex (Male)**	**3.03 (1.06, 8.70)**	**4.24**	**0.039***
Age (Years)	1.03 (0.97, 1.10)	0.87	0.352
Counterbalance	1.93 (0.70, 5.36)	1.59	0.207
**PIQ**	**0.96 (0.93, 0.99)**	**5.58**	**0.018***
**Warm *GMD***	**66.56 (10.07, 439.9)**	**18.99**	**< 0.001***
**Model Fit**	$\chi^2(6) = 50.09$	$p < 0.001$*	$R^2 = 0.591$

Cool Step 3: Best-subset Model with GMD

Predictor	aOR (95% CI)	Wald χ^2	P
Diagnosis (ASD)	1.61 (0.61, 4.26)	0.91	0.341
**Sex (Male)**	**2.83 (1.05, 7.63)**	**4.22**	**0.040***
**Age (Years)**	**1.09 (1.01, 1.16)**	**5.51**	**0.019***
Counterbalance	1.97 (0.73, 5.28)	1.81	0.179
**PIQ**	**0.95 (0.92, 0.98)**	**10.60**	**0.001***
**Cool *GMD***	**2.32 (1.07, 5.04)**	**4.55**	**0.033***
**Model Fit**	$\chi^2(6) = 28.20$	$p < 0.001$*	$R^2 = 0.396$

Note. Significant predictors in each model are bolded; PIQ = Performance IQ; GMD = Gini's Mean Difference
* $p < 0.05$

Supplementary Table S6. Regression models for warm and cool detection thresholds – Children/adolescents only

**Warm Step 1: Baseline Model**				**Cool Step 1: Baseline Model**			
**Predictor**	**aOR (95% CI)**	**Wald χ^2**	***P***	**Predictor**	**aOR (95% CI)**	**Wald χ^2**	***P***
Diagnosis (ASD)	1.60 (0.89, 2.86)	0.51	0.476	Diagnosis (ASD)	1.94 (0.89, 4.21)	2.80	0.094
Sex (Male)	1.90 (0.98, 3.70)	2.56	0.109	Sex (Male)	1.76 (0.71, 4.36)	1.49	0.223
Age (Years)	1.00 (0.97, 1.02)	1.06	0.304	**Age (Years)**	**0.88 (0.78, 0.99)**	**4.74**	**0.029***
Counterbalance	1.26 (0.71, 2.24)	0.53	0.465	Counterbalance	1.36 (0.64, 2.90)	0.63	0.429
**Model Fit**	$\chi^2(4) = 3.76$	$p = 0.440$	$R^2 = 0.043$	**Model Fit**	$\chi^2(4) = 7.50$	$p = 0.112$	$R^2 = 0.084$
**Warm Step 2: Best-subset Regression Model**				**Cool Step 2: Best-subset Regression Model**			
**Predictor**	**aOR (95% CI)**	**Wald χ^2**	***P***	**Predictor**	**aOR (95% CI)**	**Wald χ^2**	***P***
Diagnosis (ASD)	1.70 (0.58, 5.04)	0.92	0.337	Diagnosis (ASD)	2.88 (0.84, 9.85)	2.83	0.093
**Sex (Male)**	**4.16 (1.43, 12.06)**	**6.88**	**0.009***	**Sex (Male)**	**4.19 (1.44, 12.20)**	**6.93**	**0.008***
Age (Years)	0.92 (0.81, 1.04)	1.89	0.170	**Age (Years)**	**0.84 (0.74, 0.96)**	**6.73**	**0.009***
Counterbalance	1.00 (0.43, 2.29)	< 0.01	0.992	Counterbalance	1.01 (0.43, 2.39)	< 0.01	0.977
**PIQ**	**0.97 (0.94, 0.99)**	**5.85**	**0.016***	**PIQ**	**0.96 (0.93, 0.98)**	**10.91**	**0.001***
SP Low Registration	1.03 (0.98, 1.08)	1.02	0.311	SP Low Registration	1.04 (0.98, 1.09)	1.68	0.195
**Model Fit**	$\chi^2(6) = 10.81$	$p = 0.095$	$R^2 = 0.136$	**Model Fit**	$\chi^2(6) = 20.51$	$p = 0.002$*	$R^2 = 0.242$
**Warm Step 3: Best-subset Model with GMD**				**Cool Step 3: Best-subset Model with GMD**			
**Predictor**	**aOR (95% CI)**	**Wald χ^2**	***P***	**Predictor**	**aOR (95% CI)**	**Wald χ^2**	***P***
Diagnosis (ASD)	0.92 (0.30, 2.82)	0.05	0.882	Diagnosis (ASD)	1.14 (0.33, 4.00)	0.04	0.837
Sex (Male)	1.85 (0.65, 5.26)	2.11	0.247	Sex (Male)	1.57 (0.51, 4.83)	0.62	0.431
Age (Years)	0.98 (0.86, 1.12)	0.06	0.784	Age (Years)	0.99 (0.86, 1.13)	0.02	0.883
Counterbalance	1.28 (0.55, 2.99)	0.10	0.563	Counterbalance	1.38 (0.57, 3.31)	0.52	0.473
PIQ	1.00 (0.97, 1.02)	0.35	0.788	PIQ	1.00 0.97, 1.02)	0.11	0.745
SP Low Registration	1.00 (0.95, 1.05)	0.01	0.905	SP Low Registration	0.99 (0.94, 1.05)	0.13	0.721
**Warm *GMD***	**11.76 (4.32, 32.03)**	**23.24**	**< 0.001***	**Cool *GMD***	**12.58 (5.19, 30.50)**	**31.40**	**<0.001***
**Model Fit**	$\chi^2(7) = 36.16$	$p < 0.001$*	$R^2 = 0.387$	**Model Fit**	$\chi^2(7) = 59.56$	$p < 0.001$*	$R^2 = 0.553$

Note. Significant predictors in each model are bolded; PIQ = Performance IQ; SP = Sensory Profile; GMD = Gini's Mean Difference
* $p < 0.05$

Supplementary Table S7. Regression models for warm and cool detection thresholds – ASD only

Warm Step 1: Baseline Model

Predictor	aOR (95% CI)	Wald χ^2	P
Sex (Male)	1.82 (0.74, 4.50)	3.60	0.058
Age (Years)	1.01 (0.98, 1.05)	0.59	0.444
Counterbalance	1.79 (0.83, 3.84)	0.63	0.136
**Model Fit**	$\chi^2(3) = 4.15$	$p = 0.246$	$R^2 = 0.049$

Cool Step 1: Baseline Model

Predictor	aOR (95% CI)	Wald χ^2	P
Sex (Male)	1.75 (0.73, 4.18)	1.57	0.210
Age (Years)	0.99 (0.95, 1.03)	0.25	0.614
Counterbalance	1.78 (0.83, 3.83)	2.19	0.139
**Model Fit**	$\chi^2(3) = 9.61$	$p = 0.048$*	$R^2 = 0.065$

Warm Step 2: Best-subset Regression Model

Predictor	aOR (95% CI)	Wald χ^2	P
**Sex (Male)**	**2.80 (1.10, 7.12)**	**4.66**	**0.031***
Age (Years)	1.03 (0.99, 1.07)	2.44	0.118
Counterbalance	1.73 (0.81, 3.70)	2.00	0.157
**PIQ**	**0.96 (0.94, 0.99)**	**9.57**	**0.002***
**Model Fit**	$\chi^2(4) = 14.11$	$p = 0.007$*	$R^2 = 0.156$

Cool Step 2: Best-subset Regression Model

Predictor	aOR (95% CI)	Wald χ2	P
**Sex (Male)**	**2.67 (1.07, 6.68)**	**4.40**	**0.036***
Age (Years)	1.00 (0.96, 1.04)	0.04	0.845
Counterbalance	1.74 (0.81, 3.77)	1.99	0.158
**PIQ**	**0.96 (0.93, 0.98)**	**12.01**	**0.001***
**Model Fit**	$\chi^2(4) = 16.85$	$p = 0.002$*	$R^2 = 0.184$

Warm Step 3: Best-subset Model with GMD

Predictor	aOR (95% CI)	Wald χ^2	P
Sex (Male)	2.38 (0.96, 5.91)	3.48	0.062
**Age (Years)**	**1.05 (1.01, 1.09)**	**6.23**	**0.013***
Counterbalance	1.69 (0.79, 3.60)	1.85	0.174
PIQ	0.98 (0.96, 1.00)	2.60	0.107
**Warm GMD**	**2.71 (1.76, 4.19)**	**45.31**	**< 0.001***
**Model Fit**	$\chi^2(5) = 37.14$	$p < 0.001$*	$R^2 = 0.361$

Cool Step 3: Best-subset Model with GMD

Predictor	aOR (95% CI)	Wald χ^2	P
Sex (Male)	1.66 (0.65, 4.21)	1.13	0.287
Age (Years)	1.01 (0.98, 1.05)	0.51	0.475
**Counterbalance**	**2.29 (1.08, 4.89)**	**4.61**	**0.032***
**PIQ**	**0.98 (0.95, 1.00)**	**3.92**	**0.048***
**Cool GMD**	**6.64 (3.49, 12.61)**	**47.85**	**<0.001***
**Model Fit**	$\chi^2(5) = 58.40$	$p < 0.001$*	$R^2 = 0.505$

Note. Significant predictors in each model are bolded; PIQ = Performance IQ; GMD = Gini's Mean Difference
* $p < 0.05$

Supplementary Table S8. BIC weights and predictors for top five best-fitting models – Whole sample

Warm Threshold Models

Model Rank	Baseline	VIQ	PIQ	SRS T-score	SRS Item 42	BIC	ΔBIC	W_{BIC}
**1**	**X**		X			2106.09	0.00	0.490
**2**	**X**	X	X			2107.53	1.44	0.239
**3**	**X**		X	X		2109.61	3.52	0.084
**4**	**X**	**X**	**X**	**X**		2110.74	4.65	0.048
**5**	**X**					2110.82	4.73	0.046
**Predictor BIC Weight**		0.314	**0.933**	0.153	0.08			
**Predictor Evidence Ratio**		0.458	**13.977**	0.181	0.087			

Cold Threshold Models

Model Rank	Baseline	VIQ	PIQ	SRS T-score	SRS Item 42	BIC	ΔBIC	W_{BIC}
**1**	**X**		X			2096.97	0.00	0.557
**2**	**X**		X	X		2099.03	2.05	0.200
**3**	**X**	**X**	**X**			2100.27	3.30	0.107
**4**	**X**		X		X	2101.71	4.74	0.052
**5**	**X**	X	X	X		2102.27	5.30	0.039
**Predictor BIC Weight**		0.161	**0.997**	0.273	0.095			
**Predictor Evidence Ratio**		0.192	**392.704**	0.375	0.105			

Note. Weights of predictors included in the best-fitting model are bolded. BIC = Bayesian Information Criterion (Schwartz, 1978); ΔBIC = change in BIC from best-fitting model; W_{BIC} = BIC weight (Wagenmakers & Farrell, 2004); VIQ = Verbal IQ; PIQ = Performance IQ; SRS = Social Responsiveness Scale

Supplementary Table S9. BIC weights and predictors for top five best-fitting models – Adults only

Warm Threshold Models

Model Rank	Baseline	VIQ	PIQ	SRS T-score	SRS Item 42	Low Registration	Sensory Seeking	Sensory Sensitivity	BIC	ΔBIC	W_{BIC}
1	X		X						662.27	0.00	0.223
2	X		X			X			662.55	0.28	0.194
3	X	X	X						664.24	1.98	0.083
4	X		X	X					665.28	3.01	0.050
5	X		X	X		X			665.36	3.09	0.048
**Predictor BIC Weight**		0.240	**0.997**	0.148	0.143	0.413	0.121	0.138			
**Predictor Evidence Ratio**		0.316	**391.744**	0.174	0.166	0.703	0.138	0.160			

Cold Threshold Models

Model Rank	Baseline	VIQ	PIQ	SRS T-score	SRS Item 42	Low Registration	Sensory Seeking	Sensory Sensitivity	BIC	ΔBIC	W_{BIC}
1	X		X						662.14	0.00	0.314
2	X	X	X						663.73	1.59	0.142
3	X		X			X			664.93	2.80	0.078
4	X		X	X					665.39	3.25	0.062
5	X		X				X		665.90	3.76	0.048
**Predictor BIC Weight**		0.298	**0.998**	0.156	0.125	0.193	0.127	0.128			
**Predictor Evidence Ratio**		0.425	**599.832**	0.184	0.143	0.239	0.145	0.147			

Note. Weights of predictors included in the best-fitting model are bolded. Sensory quadrant scores (Low Registration, Sensory Seeking, Sensory Sensitivity, Sensory Avoiding) are based on AASP scales. BIC = Bayesian Information Criterion (Schwartz, 1978); ΔBIC = change in BIC from best-fitting model; W_{BIC} = BIC weight (Wagenmakers & Farrell, 2004); VIQ = Verbal IQ; PIQ = Performance IQ; SRS = Social Responsiveness Scale

Supplementary Table S10. BIC weights and predictors for top five best-fitting models – Children/adolescents only

Warm Threshold Models

Model Rank	Baseline	VIQ	PIQ	SRS T-score	SRS Item 42	Low Registration	Sensory Seeking	Sensory Sensitivity	BIC	ΔBIC	W_{BIC}
1	X		X			X			966.24	0.00	0.296
2	X					X			967.84	1.60	0.133
3	X		X			X		X	968.66	2.43	0.088
4	X		X	X					969.43	3.19	0.060
5	X		X	X		X			969.68	3.44	0.053
	**Predictor BIC Weight**	0.154	**0.691**	0.122	0.107	**> 0.999**	0.275	0.141			
	**Predictor Evidence Ratio**	0.183	**2.230**	0.138	0.119	**8.279E+41**	0.379	0.164			

Cold Threshold Models

Model Rank	Baseline	VIQ	PIQ	SRS T-score	SRS Item 42	Low Registration	Sensory Seeking	Sensory Sensitivity	BIC	ΔBIC	W_{BIC}
1	X		X			X			956.82	0.00	0.513
2	X		X			X		X	960.38	3.56	0.086
3	X		X			X	X		960.84	4.02	0.069
4	X		X		X	X			961.01	4.19	0.063
5	X		X	X		X			961.10	4.27	0.061
	**Predictor BIC Weight**	0.111	**0.963**	0.105	0.109	**> 0.999**	0.128	0.154			
	**Predictor Evidence Ratio**	0.125	**25.755**	0.118	0.122	**2.768E+42**	0.147	0.182			

Note. Weights of predictors included in the best-fitting model are bolded. Sensory quadrant scores (Low Registration, Sensory Seeking, Sensory Sensitivity, Sensory Avoiding) are based on SP caregiver scales. BIC = Bayesian Information Criterion (Schwartz, 1978); ΔBIC = change in BIC from best-fitting model; W_{BIC} = BIC weight (Wagenmakers & Farrell, 2004); VIQ = Verbal IQ; PIQ = Performance IQ; SRS = Social Responsiveness Scale

Supplementary Table S11. BIC weights and predictors for top five best-fitting models – ASD only

Warm Threshold Models

Model Rank	Baseline	VIQ	PIQ	SRS T-score	SRS Item 42	ADOS CSS	Any Psych Med	SSRI	Stimulant	BIC	ΔBIC	W_{BIC}
1	X		X							1091.92	0.00	0.194
2	X	X	X							1092.67	0.75	0.134
3	X		X				X			1094.74	2.82	0.047
4	X	X	X					X		1094.90	2.97	0.044
5	X		X				X	X		1095.24	3.32	0.037
**Predictor BIC Weight**		0.409	**0.960**	0.105	0.115	0.201	0.241	0.271	0.137			
**Predictor Evidence Ratio**		0.693	**23.878**	0.117	0.130	0.251	0.318	0.372	0.158			

Cold Threshold Models

Model Rank	Baseline	VIQ	PIQ	SRS T-score	SRS Item 42	ADOS CSS	Any Psych Med	SSRI	Stimulant	BIC	ΔBIC	W_{BIC}
1	X		X							662.14	0.00	0.314
2	X		X			X				663.73	1.59	0.142
3	X		X	X		X				664.93	2.80	0.078
4	X	X	X			X				665.39	3.25	0.062
5	X		X					X		665.90	3.76	0.048
**Predictor BIC Weight**		0.182	**0.991**	0.210	0.102	0.552	0.146	0.173	0.107			
**Predictor Evidence Ratio**		0.222	**39.078**	0.266	0.113	1.230	0.170	0.209	0.119			

Note. Weights of predictors included in the best-fitting model are bolded. BIC = Bayesian Information Criterion (Schwartz, 1978); ΔBIC = change in BIC from best-fitting model; W_{BIC} = BIC weight (Wagenmakers & Farrell, 2004); VIQ = Verbal IQ; PIQ = Performance IQ; SRS = Social Responsiveness Scale; ADOS CSS = Autism Diagnostic Observation Schedule–2nd edition Calibrated Severity Score

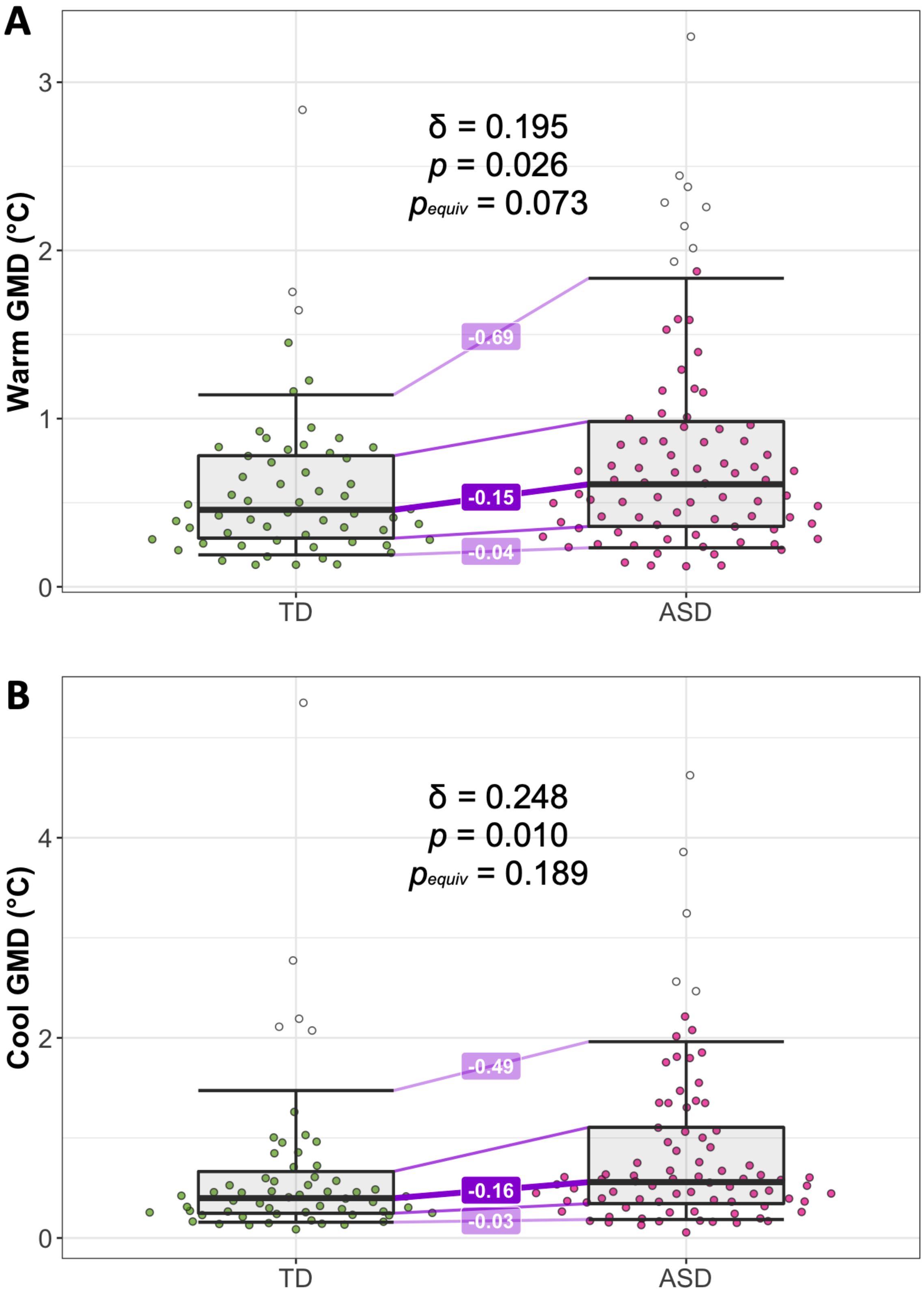

Supplementary Figure S1. Comparison of GMD values between the two diagnostic groups.
(**A**) Warm trial *GMD* values based on $n = 10$ trials per subject. (**B**) Cool trial *GMD* values based on $n = 10$ trials per subject. Horizontal lines are not typical boxplot marks but instead represent the 0.1, 0.25, 0.5, 0.75, and 0.9 Harrell-Davis quantiles of each group distribution. Differences in group quantiles (TD – ASD) are depicted as lines bridging the two groups. Outliers (defined by applying the boxplot rule to each group distribution) are represented as unshaded points.

Supplementary Methods

Participants

Thirty-three adults with ASD and 24 adults with typical development (TD) participated in the study. Individuals in the ASD group were excluded if they reported any comorbid psychopathology within the last five years other than anxiety disorders or ADHD. No restrictions were placed on the psychotropic medications taken at the time of the study. Diagnoses of ASD were confirmed through research-reliable administrations of the Autism Diagnostic Observation Schedule—Second Edition (ADOS-2; Lord et al., 2012) by a licensed clinical psychologist specializing in the assessment of ASD. The definitive judgment of diagnostic status was made based on the clinical judgment of the licensed clinical psychologist, guided by her time spent administering the ADOS-2 and cognitive tests to the participant. Individuals in the TD group were excluded if they had a history of psychiatric disorder, cognitive impairment, sensory impairments, psychotropic medication use, or clinically elevated scores on the Social Responsiveness Scale–Second Edition (SRS-2; Constantino & Gruber, 2012) or the Adult Self Report (ASR; Achenbach & Rescorla, 2003). Participants in both groups were excluded for genetic and neurological disorders, significant head injuries, or any sensory impairments with known etiology other than ASD. One ASD participant was found to have a full-scale IQ of 63 and was subsequently excluded from all analyses. An additional two participants in the ASD group scored below the diagnostic cutoff on the ADOS-2, scoring one and two points below the cutoff, respectively. However, the examining psychologist felt that in both cases, these individuals met DSM–5 ASD criteria based on her clinical interview, and they were thus retained for analysis. The final sample of adults (Table 1 of main article) contained 32 participants with ASD (21 male, median age 25.50 years) and 24 with TD (14 male, median age 29.76 years).

In total, 51 children and adolescents with ASD and 36 children and adolescents with TD, ages 7–17 years, participated in the study. Inclusion criteria for children and adolescents were similar to those for adults, with several exceptions. ASD diagnoses were supported by scores from the ADOS-2, and in a subset of children, the Autism Diagnostic Interview-Revised (ADI-R; Lord, Rutter, & Le Couteur, 1994). Parents who were not able to complete the full ADI-R received a semi-structured clinical interview from the psychologist

that focused on developmental history and utilized a number of the ADI-R prompts. As with the adult sample, the definitive judgment of diagnostic status was made based on the clinical judgment of the licensed clinical psychologist, guided, but not constrained by ADOS and/or ADI-R scores. Additionally, children and adolescents in the ASD group were only excluded for comorbid psychopathology (beyond anxiety and ADHD) that required treatment within the previous two years. In the TD group, the parent-report Child Behavior Checklist (CBCL; Achenbach & Rescorla, 2001) was used as a screening measure for psychopathology. Parents of TD individuals were additionally asked to complete the Social Communication Questionnaire (SCQ; Rutter, Bailey, & Lord, 2003), with scores of 15 or lower required to participate in the study. One adolescent in the TD group was excluded due to a parent report of elevated autistic traits (SRS-2 total T-score = 76). The final child and adolescent sample (Table 1) consisted of 51 participants with ASD (41 male, median age 10.03 years) and 35 participants with TD (26 male, mean age 9.21 years).

Measures

Social Responsiveness Scale–Second Edition The Social Responsiveness Scale–Second Edition (SRS-2; Constantino & Gruber, 2012) is a widely-used measure of quantitative autistic traits in both the general population and individuals with ASD (Bruni, 2014). The original SRS (Constantino & Todd, 2000; Constantino et al., 2000; 2003), now termed the SRS-2 School Age Form but otherwise unchanged, measures autistic traits in children 4–18 years of age via parent report. Total scores on the SRS-2 range from 0–195, with higher scores indicating higher levels of autistic symptomatology. Items are also organized into theoretically-derived subscales, but psychometric evidence to date has not supported the separate nature of these subconstructs (Bölte et al., 2008; Constantino, 2011; Frazier et al., 2014; Sturm et al., 2017), leading many to utilize only the total scale score as a measure of general autistic symptomatology. With the development of the SRS-2, parallel forms for preschoolers and adults were released with questions rephrased to apply to those populations. The SRS-2 adult form is available as either a self-report or caregiver-report form. T-scores (M = 50, SD = 10) are also available for individuals based on sex and the specific form used.

Psychometric properties of the various SRS-2 form total scores, including internal consistency, test-retest reliability, inter-rater reliability, content validity, construct validity, and concurrent validity with other ASD trait measures and clinical diagnoses have all been examined and are purported to be generally strong across a number of published studies (Bruni, 2014). Cross-cultural adaptations have also been undertaken with strong psychometrics in those samples as well (Bölte, 2008; 2012; Gau et al., 2013; Nishiyama et al., 2014; Takei et al., 2014; Wang et al., 2012, Wigham et al., 2012). Of note, the SRS-2 total score is confounded to some extent by general psychopathology in both ASD probands and the general population (Hus et al., 2013; Nishiyama et al., 2014), and the specificity of high SRS score for ASD diagnosis compared to several other psychiatric conditions is substantially lower than when discriminating ASD from the general population (Cholemkery et al., 2014a; 2014b; Darrow et al., 2017; Matsuo et al., 2015; Moul et al., 2015; Pine et al., 2008; Stewart et al., 2016b; Solomon et al., 2011; South, 2017; Takei et al., 2014). Thus, when considering the interpretation of SRS-2 scores, it is likely that higher scores reflect a combination of both ASD-specific traits and more general psychopathology.

In the current study, individuals in the child/adolescent group were rated by a parent or primary caregiver using the SRS-2 School Age Form. Adult participants completed the SRS-2 Adult Self-Report Form. SRS-2 T-Scores based on the total score were calculated for all participants and used as dimensional measures of autistic traits in further analyses. Additionally, scores on SRS-2 item 42 (Self-report: *I am overly sensitive to certain sounds, textures, or smells*; Caregiver-report: *Seems overly sensitive to certain sounds, textures, or smells*) were additionally included in analyses as a one-item measure of sensory hyperresponsiveness.

Sensory Profile The Sensory Profile (SP; Dunn, 1999) is a caregiver questionnaire that assesses the frequency of a large number of behaviors theoretically related to the child's sensory experiences. The questionnaire is based on the conceptual model of Dunn (1997, 2001), wherein the combination of sensory threshold (high or low) and behavioral response (passive or active) generates four theoretical sensory quadrants: Low Registration (Low, Passive), Sensory Seeking (Low, Active), Sensory Sensitivity (High, Passive), and Sensory Avoiding (High, Active). The SP generates scores for each of the four quadrants, as well as modality-specific scores. Psychometric properties of quadrant scores, including internal consistency, test-retest reliability,

and discriminant validity between controls and children with sensory processing difficulties have been reported as adequate to good (cf. Jorquera-Cabrera et al., 2017; Ohl et al., 2012), although no study to date has assessed the psychometric properties of the full-length SP in individuals with ASD (see also Williams et al., 2018 for a psychometric analysis of the Short Sensory Profile in ASD). Nevertheless, the SP and its shortened form the Short Sensory Profile (SSP) are by far the most commonly utilized measures of this sort for individuals with ASD (Burns et al., 2017). Scores on all SP scales, including the quadrant scores, have been found to discriminate individuals with ASD from both healthy controls and their unaffected siblings (Hilton et al., 2016).

Caregivers of participants in our child/adolescent group filled out the SP, from which the four quadrant scores were extracted for use in analyses. Of these, only the Low Registration, Sensory Seeking and Sensory Sensitivity scales were utilized as potential predictors in regression models due to the large correlations between the Sensory Avoiding subscale and two of the other SP subscales in our sample (Sensory Sensitivity: Spearman's $r = 0.765$; Low Registration: Spearman's $r = 0.860$). The SP also contains a single item, *Decreased awareness of pain and temperature*, which directly assesses the behaviors alluded to in the DSM-5 criteria. However, this item was not heavily endorsed in our sample (only 9 of 74 caregivers, all of whom had children with ASD, reported this behavior as occurring "Always" or "Frequently"), and thus was not expected to explain significant variance in the regression models. Exploratory correlation analyses were still carried out to assess the relationships between this one-item scale and warm and cool detection thresholds in the children whose caregivers supplied this data. Notably, much of the child/adolescent group was older than 10 years of age, the upper limit of the SP age ranged used for the normative sample. However, due to the historical paucity of sensory measures in ASD research, researchers including the author of the measure have used SP and SSP scores in combined child/adolescent samples with ASD (e.g., Ermer & Dunn, 1998; Gabriels et al., 2008; Green et al., 2016, Kern et al., 2007; Kientz & Dunn, 1997; Lane et al., 2014; Schoen et al., 2009; Uljarevic et al., 2016).

Adolescent/Adult Sensory Profile The Adolescent/Adult Sensory Profile (AASP; Brown et al., 2001; Brown & Dunn, 2002) is a self-report questionnaire that assesses a range of attitudes and behaviors theoretically related to sensory processing in individuals 11 years and older. Like the SP, the AASP is organized into

subscales based on the four quadrants of Dunn's (1997, 2001) theoretical model. AASP total scores ranges from 60 to 300 with individual quadrant scores ranging from 15 to 75. Psychometric properties of the AASP are less well studied, with the original authors (Brown & Dunn, 2002) reporting questionable to acceptable internal consistency estimates for quadrant scores (coefficient alpha = 0.64–0.78). Strong test-retest reliabilities (ICC(3,2) = 0.76–0.88) for the four quadrant scores were found in a sample of older adults using a Chinese translation of the AASP.

Like the caregiver SP, the AASP is the most popular sensory measure in studies of adults with ASD (Burns et al., 2017; DuBois et al., 2017) despite its psychometric properties not being investigated in the ASD population to date. Nevertheless, there is substantial indirect evidence to support the potential usefulness of the AASP in ASD. Small to moderate ASD–TD group differences have been reported in AASP quadrant scores (higher scores in ASD for Low Registration, Sensory Sensitivity, and Sensory Avoiding, lower scores for Sensory Seeking; Crane et al., 2009; De la Marche et al., 2012; Mayer, 2017). The same pattern of AASP scores is also exhibited in parents of individuals with ASD (Donaldson et al., 2016; Uljarevic et al., 2014), with parents from multiplex ASD families reporting more sensory atypicalities than those from simplex ASD families (Donaldson et al., 2016). The AASP total score and quadrants also exhibit moderate to strong correlations with ASD trait measures and other measures of sensory features in general population and ASD samples (Horder et al., 2014; Mayer, 2017). Some relationships have been found between AASP scores and more objective task-based measures of sensory abnormality in ASD, but methodologies are extremely varied, and these relationships have been highly inconsistent across studies (e.g., Fukuyama et al., 2017; Jones et al., 2009; Karhson & Golob, 2016; Keehn et al., 2017; Stewart et al., 2016a; Takarae et al., 2016).

Participants in the adult group completed the AASP, from which the four quadrant scores were extracted for analysis. Of these, only the Low Registration, Sensory Seeking and Sensory Sensitivity scales were utilized as potential predictors in regression models due to the large correlations between the Sensory Sensitivity and Sensory Avoiding subscales in our sample (Spearman's $r = 0.868$).

ADOS-2 The Autism Diagnostic Observation Schedule–Second Edition (ADOS-2; Lord et al., 2012) is a structured clinician-administered assessment of autism features typically used to establish a diagnosis of ASD.

The ADOS-2 is administered as one of multiple modules appropriate for different subsets of the ASD population based on age and language ability. The items assessed on the ADOS-2 are identical to those on the previous version of the ADOS (Lord et al., 2000), with different combinations of items being utilized in the updated diagnostic algorithm (Gotham et al., 2007, 2008; Hus & Lord, 2014; Pugliese et al., 2015). The ADOS-2 modules each provide a total score, as well as subscale scores for the items reflecting the two DSM-congruent ASD domains of social affect (SA) and restricted/repetitive behaviors (RRB). Because ADOS-2 total and subscale raw scores are not directly comparable across modules, the authors have developed calibrated severity scores (CSS; Bal & Lord, 2015; Gotham et al., 2009; Hus et al., 2014; Hus & Lord, 2014), which allow ADOS-2 total and subscale scores from all modules to be compared on a common 1–10 metric that is minimally related to age and IQ. The psychometric properties of the ADOS-2 have been extensively studied, with generally strong performance in ASD diagnostic classification (Dorlack et al., 2018). Consistency across raters is dependent on the experience of the clinician with the measure, but in those who are trained to research reliability, agreement across items and diagnosis is good (Kamp-Becker et al., 2018). Rigorous item response theory analyses have been conducted on modules 3 and 4, indicating that ADOS-2 measures general ASD severity and SA severity very reliably when individuals scores are close to the diagnostic threshold (Kuhfeld & Sturm, 2018). The same analysis indicated that the ADOS-2 RRB scores were not very reliable across the entire continuum of severity and cautioned against the use of the ADOS-2 RRB score as a measure of RRB severity. ADOS scores, once converted to the CSS, are stable across time (Bieleninik et al., 2017), independent of sex (Tillmann et al., 2018), and less confounded by age and cognitive ability than raw scores (Bal & Lord, 2015; Shumway et al., 2012).

ASD participants in our sample were administered the ADOS-2 module 3 or 4, based on age and developmental level, by a licensed clinical psychologist trained to research reliability on the measure. Raw total scores were extracted and converted to overall CSS, which were then used as measures of ASD severity in further analyses. Because of recent findings questioning the reliability of ADOS-2 RRB scores (Kuhfeld & Sturm, 2018), we chose not to utilize the separate SA and RRB CSS as predictors in our regression models.

Missing Data Although the majority of participants completed all relevant measures in the experimental protocol, usable data was not present for all participants for the SRS-2, WASI-II, SP, AASP, and ADOS-2 (*M* =

14.6% missing cases). The exact numbers of participants completing each measure is present in Table 2 of the main article. Reasons for missing data were multifactorial, including variability in participant compliance with the questionnaire battery and experimenter errors in fidelity procedures such as checking the completeness of each form. With regard to the ADOS-2 in particular, a number of participants originally completed this measure as a part of a separate study at our institution, and their scores on this measure were shared with our lab group. However, as some of the participants' scores were recorded using the original ADOS diagnostic algorithm, not all of these scores contained enough item-level information to calculate the ADOS-2 algorithm scores and corresponding calibrated severity scores.

Ordinal Logistic Regression Models

In order to determine the effects of various predictor variables on thermal thresholds, we conducted a multiple regression analysis. Due to the distributional properties of thermal threshold data outlined in the introduction, we believed that a rank-based method would be most appropriate for analyses. Thus, we chose to conduct an ordinal logistic regression using the cumulative probability model with logit link function (CPM; Harrell, 2015; Liu et al., 2017), which is appropriate for use with continuous outcomes. The CPM is a semi-parametric regression model that functions as a multi-predictor generalization of the Wilcoxon–Mann–Whitney test. As with standard logistic regression models, predictors are interpreted as odds ratios, in this case associated with the odds that the outcome variable will be associated with a higher value of the outcome versus a lower value. See Liu et al., 2017 for the full mathematical description of the model and in-depth discussion of its statistical properties.

The significance of the model as a whole is tested using a likelihood ratio χ^2 test that compares the fully-specified model to a baseline model that includes only intercept terms. The quantile-quantile plot of probability scale residuals (Li & Shepherd, 2012; Shepherd, Li, & Liu, 2016) versus a uniform distribution was also examined. In addition, several other measures of fit are available for this model, including the Bayesian Information Criterion (BIC; Schwartz, 1978) and Nagelkerke (1991) R^2 index were considered. Further indices available to describe these models are discussed in Agresti & Tarantola (2018).

Regression models for the thermal threshold variables were fit in three steps: (1) baseline model (diagnostic group, age, sex, and counterbalance order), (2) best-subset regression using the Bayesian Information Criterion (BIC; Schwarz, 1978; Gagné & Dayton, 2002), (3) add Gini's Mean Difference (*GMD*) to the final model. The best-subset regression included the following predictors: Verbal IQ, Performance IQ, SRS-2 T-score, and SRS-2 sensory item (item 42) score. Additional variables were included in the models that were restricted to only a subset of the study sample: three SP quadrant scores (Low Registration, Sensory Sensitivity, Sensory Seeking; child/adolescent group only), three AASP quadrant scores (Low Registration, Sensory Sensitivity, Sensory Seeking; adult group only), ADOS-2 overall CSS (ASD group only), use of any psychiatric medication (ASD group only), use of an SSRI (ASD group only), and use of a psychostimulant (ASD group only).

During the best-subset regression step, BIC weights (Wagenmakers & Farrell, 2004) were used to quantify the probability that the chosen model was the best model, the superiority of the best-fitting model over the closest competitor and baseline models, and the probability that each predictor is included in the best model. To calculate the BIC weight for a model, all possible models in the best-subset regression are fit and their respective BIC values are calculated. The ΔBIC for each model is then calculated by subtracting the BIC value of the best candidate model (i.e., the minimum BIC value in the set) from the BIC values of all other models. An estimate of the relative likelihood L of model i may then be obtained by the simple transform (Wagenmakers & Farrell, 2004, equation 3):

$$L(M_i|\text{data}) \propto \exp(-\frac{1}{2}\Delta\text{BIC}_i)$$

By normalizing the relative likelihoods (i.e., dividing the relative likelihoods by the sum of relative likelihoods for all models such that the normalized values sum to 1), one is able to calculate the BIC weight, $w_i(\text{BIC})$, interpretable as the probability that M_i is the best model among the set of K candidate models, given the data.

$$w_i(\text{BIC}) = \frac{\exp(-\frac{1}{2}\Delta\text{BIC}_i)}{\sum_{k=1}^{K}\exp(-\frac{1}{2}\Delta\text{BIC}_k)}$$

Once calculated, the BIC weights can be further used to compare models or groups of models against each other. To compare two models, one simply divides their BIC weights, resulting in the evidence ratio, the strength of evidence in favor of one model over the other. One may also calculate the evidence ratio for a particular predictor term by summing the BIC weights for all models that include the predictor variable and dividing by the sum of all BIC weights that do not include that variable. Evidence ratios greater than one provide evidence for the model or set of models being tested, and larger values represent larger amounts of evidence.

References

Achenbach, T. M., & Rescorla, L. (2001). ASEBA school-age forms & profiles.

Achenbach, T. M., & Rescorla, L. (2003). *Manual for the ASEBA adult forms & profiles: for ages 18-59: adult self-report and adult behavior checklist*. ASEBA.

Agresti, A., & Tarantola, C. (2018). Simple ways to interpret effects in modeling ordinal categorical data. *Statistica Neerlandica*, *72*(3), 210–223. http://doi.org/10.1111/stan.12130

Hus Bal, V., & Lord, C. (2015). Replication of Standardized ADOS Domain Scores in the Simons Simplex Collection. *Autism Research*, *8*(5), 583–592. http://doi.org/10.1002/aur.1474

Bieleninik, Ł., Posserud, M.-B., Geretsegger, M., Thompson, G., Elefant, C., & Gold, C. (2017). Tracing the temporal stability of autism spectrum diagnosis and severity as measured by the Autism Diagnostic Observation Schedule: A systematic review and meta-analysis. *PloS One*, *12*(9), 1–23. http://doi.org/10.1371/journal.pone.0183160

Bölte, S. (2012). Brief Report: the Social Responsiveness Scale for Adults (SRS-A): initial results in a German cohort. *Journal of Autism and Developmental Disorders*, *42*(9), 1998–1999. http://doi.org/10.1007/s10803-011-1424-5

Bölte, S., Poustka, F., & Constantino, J. N. (2008). Assessing autistic traits: cross-cultural validation of the social responsiveness scale (SRS). *Autism Research*, *1*(6), 354–363. http://doi.org/10.1002/aur.49

Brown, C., Tollefson, N., Dunn, W., Cromwell, R., & Filion, D. (2001). The Adult Sensory Profile: measuring patterns of sensory processing. *American Journal of Occupational Therapy*, *55*(1), 75–82.

Brown, C., & Dunn, W. (2002). *Adolescent-adult sensory profile: user's manual*. San Antonio: Therapy Skill Builders.

Bruni, T. P. (2014). Test Review: Social Responsiveness Scale–Second Edition (SRS-2). *Journal of Psychoeducational Assessment*, *32*(4), 365–369. http://doi.org/10.1177/0734282913517525

Burns, C. O., Dixon, D. R., Novack, M., & Granpeesheh, D. (2017). A Systematic Review of Assessments for Sensory Processing Abnormalities in Autism Spectrum Disorder. *Review Journal of Autism and Developmental Disorders*, *4*(3), 209–224. http://doi.org/10.1007/s40489-017-0109-1

Cholemkery, H., Mojica, L., Rohrmann, S., Gensthaler, A., & Freitag, C. M. (2014). Can Autism Spectrum Disorders and Social Anxiety Disorders be Differentiated by the Social Responsiveness Scale in Children and Adolescents? *Journal of Autism and Developmental Disorders*, *44*(5), 1168–1182. http://doi.org/10.1007/s10803-013-1979-4

Cholemkery, H., Kitzerow, J., Rohrmann, S., & Freitag, C. M. (2014). Validity of the social responsiveness scale to differentiate between autism spectrum disorders and disruptive behaviour disorders. *European Child & Adolescent Psychiatry*, *23*(2), 81–93. http://doi.org/10.1007/s00787-013-0427-5

Chung, J. C. C. (2006). Measuring sensory processing patterns of older Chinese people: Psychometric validation of the adult sensory profile. *Aging & Mental Health*, *10*(6), 648–655. http://doi.org/10.1080/13607860600648080

Constantino, J. N., Przybeck, T., Friesen, D., & Todd, R. D. (2000). Reciprocal social behavior in children with and without pervasive developmental disorders. *Journal of Developmental & Behavioral Pediatrics*, *21*(1), 2–11.

Constantino, J. N., & Todd, R. D. (2000). Genetic Structure of Reciprocal Social Behavior. *American Journal of Psychiatry*, *157*(12), 2043–2045. http://doi.org/10.1176/appi.ajp.157.12.2043

Constantino, J. N., Davis, S. A., Todd, R. D., Schindler, M. K., Gross, M. M., Brophy, S. L., et al. (2003). Validation of a Brief Quantitative Measure of Autistic Traits: Comparison of the Social Responsiveness

Scale with the Autism Diagnostic Interview-Revised. *Journal of Autism and Developmental Disorders*, *33*(4), 427–433. http://doi.org/10.1023/A:1025014929212

Constantino, J. N. (2011). The Quantitative Nature of Autistic Social Impairment. *Pediatric Research*, *69*(5 Part 2), 55R–62R. http://doi.org/10.1203/PDR.0b013e318212ec6e

Constantino, J. N. & Gruber, C. P. (2012). *Social Responsiveness Scale–Second Edition (SRS-2)*. Torrance, CA: Western Psychological Services.

Crane, L., Goddard, L., & Pring, L. (2009). Sensory processing in adults with autism spectrum disorders. *Autism*, *13*(3), 215–228. http://doi.org/10.1177/1362361309103794

Darrow, S. M., Grados, M., Sandor, P., Hirschtritt, M. E., Illmann, C., Osiecki, L., et al. (2017). Autism Spectrum Symptoms in a Tourette's Disorder Sample. *Journal of the American Academy of Child and Adolescent Psychiatry*, *56*(7), 610–617.e1. http://doi.org/10.1016/j.jaac.2017.05.002

Donaldson, C. K., Stauder, J. E. A., & Donkers, F. C. L. (2016). Increased Sensory Processing Atypicalities in Parents of Multiplex ASD Families Versus Typically Developing and Simplex ASD Families. *Journal of Autism and Developmental Disorders*, *69*(8), 357–14. http://doi.org/10.1007/s10803-016-2888-0

Dorlack, T. P., Myers, O. B., & Kodituwakku, P. W. (2018). A Comparative Analysis of the ADOS-G and ADOS-2 Algorithms: Preliminary Findings. *Journal of Autism and Developmental Disorders*, *48*(6), 2078–2089. http://doi.org/10.1007/s10803-018-3475-3

DuBois, D., Lymer, E., Gibson, B., Desarkar, P., & Nalder, E. (2017). Assessing Sensory Processing Dysfunction in Adults and Adolescents with Autism Spectrum Disorder: A Scoping Review. *Brain Sciences*, *7*(8), 108. http://doi.org/10.3390/brainsci7080108

Dunn, W. (1997). The Impact of Sensory Processing Abilities on the Daily Lives of Young Children and Their Families: A Conceptual Model. *Infants & Young Children*, *9*(4), 23–35. http://doi.org/10.1097/00001163-199704000-00005

Dunn, W. (1999). *Sensory profile: User's manual*. San Antonio, TX: Psychological Corporation.

Dunn, W. (2001). The Sensations of Everyday Life: Empirical, Theoretical, and Pragmatic Considerations. *American Journal of Occupational Therapy*, *55*(6), 608–620. http://doi.org/10.5014/ajot.55.6.608

Ermer, J., & Dunn, W. (1998). The sensory profile: a discriminant analysis of children with and without disabilities. *American Journal of Occupational Therapy*, *52*(4), 283–290.

Feng, D., & Cliff, N. (2004). Monte Carlo Evaluation of Ordinal d with Improved Confidence Interval. *Journal of Modern Applied Statistical Methods*, *3*(2), 322–332. http://doi.org/10.22237/jmasm/1099267560

Frazier, T. W., Ratliff, K. R., Gruber, C., Zhang, Y., Law, P. A., & Constantino, J. N. (2014). Confirmatory factor analytic structure and measurement invariance of quantitative autistic traits measured by the social responsiveness scale-2. *Autism*, *18*(1), 31–44. http://doi.org/10.1177/1362361313500382

Fukuyama, H., Kumagaya, S.-I., Asada, K., Ayaya, S., & Kato, M. (2017). Autonomic versus perceptual accounts for tactile hypersensitivity in autism spectrum disorder. *Scientific Reports*, *7*(1). http://doi.org/10.1038/s41598-017-08730-3

Gabriels, R. L., Agnew, J. A., Miller, L. J., Gralla, J., & Pan, Z. (2008). Is there a relationship between restricted, repetitive, stereotyped behaviors and interests and abnormal sensory response in children with autism spectrum disorders? *Research in Autism Spectrum Disorders*, *2*(4), 660–670. http://doi.org/10.1016/j.rasd.2008.02.002

Gagné, P., & Dayton, C. M. (2002). Best Regression Model Using Information Criteria. *Journal of Modern Applied Statistical Methods*, *1*(2), 479–488. http://doi.org/10.22237/jmasm/1036110180

Gau, S. S. F., Liu, L.-T., Wu, Y.-Y., Chiu, Y.-N., & Tsai, W.-C. (2013). Psychometric properties of the Chinese version of the Social Responsiveness Scale. *Research in Autism Spectrum Disorders*, *7*(2), 349–360. http://doi.org/10.1016/j.rasd.2012.10.004

Gotham, K., Pickles, A., & Lord, C. (2009). Standardizing ADOS scores for a measure of severity in autism spectrum disorders. *Journal of Autism and Developmental Disorders*, *39*(5), 693–705. http://doi.org/10.1007/s10803-008-0674-3

Gotham, K., Risi, S., Dawson, G., Tager-Flusberg, H., JOSEPH, R., Carter, A., et al. (2008). A Replication of the Autism Diagnostic Observation Schedule (ADOS) Revised Algorithms. *Journal of the American Academy of Child and Adolescent Psychiatry*, *47*(6), 642–651. http://doi.org/10.1097/CHI.0b013e31816bffb7

Gotham, K., Risi, S., Pickles, A., & Lord, C. (2007). The Autism Diagnostic Observation Schedule: revised algorithms for improved diagnostic validity. *Journal of Autism and Developmental Disorders*, *37*(4), 613–627. http://doi.org/10.1007/s10803-006-0280-1

Green, D., Chandler, S., Charman, T., Simonoff, E., & Baird, G. (2016). Brief Report: DSM-5 Sensory Behaviours in Children With and Without an Autism Spectrum Disorder. *Journal of Autism and Developmental Disorders*, *46*(11), 3597–3606. http://doi.org/10.1007/s10803-016-2881-7

Harrell, F. E., Jr. (2015). Ordinal Logistic Regression. In *Regression Modeling Strategies with Applications, to Linear Models, Logistic and Ordinal Regression, and Survival Analysis* (2nd ed., pp. 311–325). Heidelberg: Springer. http://doi.org/10.1007/978-3-319-19425-7_13

Hilton, C. L., Babb-Keeble, A., Westover, E. E., Zhang, Y., Adams, C., Collins, D. M., et al. (2016). Sensory Responsiveness in Siblings of Children with Autism Spectrum Disorders. *Journal of Autism and Developmental Disorders*, *46*(12), 3778–3787. http://doi.org/10.1007/s10803-016-2918-y

Horder, J., Wilson, C. E., Mendez, M. A., & Murphy, D. G. (2014). Autistic traits and abnormal sensory experiences in adults. *Journal of Autism and Developmental Disorders*, *44*(6), 1461–1469. http://doi.org/10.1007/s10803-013-2012-7

Hus Bal, V., & Lord, C. (2015). Replication of Standardized ADOS Domain Scores in the Simons Simplex Collection. *Autism Research*, *8*(5), 583–592. http://doi.org/10.1002/aur.1474

Hus, V., & Lord, C. (2014). The autism diagnostic observation schedule, module 4: revised algorithm and standardized severity scores. *Journal of Autism and Developmental Disorders*, *44*(8), 1996–2012. http://doi.org/10.1007/s10803-014-2080-3

Hus, V., Gotham, K., & Lord, C. (2014). Standardizing ADOS domain scores: separating severity of social affect and restricted and repetitive behaviors. *Journal of Autism and Developmental Disorders*, *44*(10), 2400–2412. http://doi.org/10.1007/s10803-012-1719-1

Hus, V., Bishop, S., Gotham, K., Huerta, M., & Lord, C. (2013). Factors influencing scores on the social responsiveness scale. *Journal of Child Psychology and Psychiatry*, *54*(2), 216–224. http://doi.org/10.1111/j.1469-7610.2012.02589.x

Jones, C. R. G., Happé, F., Baird, G., Simonoff, E., Marsden, A. J. S., Tregay, J., et al. (2009). Auditory discrimination and auditory sensory behaviours in autism spectrum disorders. *Neuropsychologia*, *47*(13), 2850–2858. http://doi.org/10.1016/j.neuropsychologia.2009.06.015

Jorquera-Cabrera, S., Romero-Ayuso, D., Rodriguez-Gil, G., & Triviño-Juárez, J.-M. (2017). Assessment of Sensory Processing Characteristics in Children between 3 and 11 Years Old: A Systematic Review. *Frontiers in Pediatrics*, *5*(2), 57. http://doi.org/10.3389/fped.2017.00057

Kamp-Becker, I., Albertowski, K., Becker, J., Ghahreman, M., Langmann, A., Mingebach, T., et al. (2018). Diagnostic accuracy of the ADOS and ADOS-2 in clinical practice. *European Child & Adolescent Psychiatry*, *15*(3), 1–15. http://doi.org/10.1007/s00787-018-1143-y

Karhson, D. S., & Golob, E. J. (2016). Atypical sensory reactivity influences auditory attentional control in adults with autism spectrum disorders. *Autism Research*, *9*(10), 1079–1092. http://doi.org/10.1002/aur.1593

Kern, J. K., Garver, C. R., Carmody, T., Andrews, A. A., Trivedi, M. H., & Mehta, J. A. (2007). Examining sensory quadrants in autism. *Research in Autism Spectrum Disorders*, *1*(2), 185–193. http://doi.org/10.1016/j.rasd.2006.09.002

Kientz, M. A., & Dunn, W. (1997). A comparison of the performance of children with and without autism on the Sensory Profile. *American Journal of Occupational Therapy*, *51*(7), 530–537.

Kuhfeld, M., & Sturm, A. (2018). An examination of the precision of the Autism Diagnostic Observation Schedule using item response theory. *Psychological Assessment*, *30*(5), 656–668. http://doi.org/10.1037/pas0000512

De la Marche, W., Steyaert, J., & Noens, I. (2012). Atypical sensory processing in adolescents with an autism spectrum disorder and their non-affected siblings. *Research in Autism Spectrum Disorders*, *6*(2), 639–645. http://doi.org/10.1016/j.rasd.2011.09.014

Keehn, R. J. J., Sanchez, S. S., Stewart, C. R., Zhao, W., Grenesko-Stevens, E. L., Keehn, B., & Müller, R.-A. (2017). Impaired downregulation of visual cortex during auditory processing is associated with autism symptomatology in children and adolescents with autism spectrum disorder. *Autism Research*, *10*(1), 130–143. http://doi.org/10.1002/aur.1636

Lane, A. E., Molloy, C. A., & Bishop, S. L. (2014). Classification of children with autism spectrum disorder by sensory subtype: a case for sensory-based phenotypes. *Autism Research*, *7*(3), 322–333. http://doi.org/10.1002/aur.1368

Li, C., & Shepherd, B. E. (2012). A new residual for ordinal outcomes. *Biometrika*, *99*(2), 473–480. http://doi.org/10.1093/biomet/asr073

Liu, Q., Shepherd, B. E., Li, C., & Harrell, F. E., Jr. (2017). Modeling continuous response variables using ordinal regression. *Statistics in Medicine*, *36*(27), 4316–4335. http://doi.org/10.1002/sim.7433

Lord, C., Rutter, M., & Le Couteur, A. (1994). Autism Diagnostic Interview-Revised: A revised version of a diagnostic interview for caregivers of individuals with possible pervasive developmental disorders. *Journal of Autism and Developmental Disorders*, *24*(5), 659–685. http://doi.org/10.1007/BF02172145

Lord, C., Risi, S., Lambrecht, L., Cook, E. H., Leventhal, B. L., DiLavore, P. C., et al. (2000). The autism diagnostic observation schedule-generic: a standard measure of social and communication deficits associated with the spectrum of autism. *Journal of Autism and Developmental Disorders*, *30*(3), 205–223.

Lord, C., Rutter, M., DiLavore, P., Risi, S., Gotham, K., & Bishop, S. L. (2012). *Autism diagnostic observation schedule (ADOS-2): manual* (2nd ed.). Los Angeles: Western Psychological Services.

Matsuo, J., Kamio, Y., Takahashi, H., Ota, M., Teraishi, T., Hori, H., et al. (2015). Autistic-like traits in adult patients with mood disorders and schizophrenia. *PloS One*, *10*(4). http://doi.org/10.1371/journal.pone.0122711

Mayer, J. L. (2017). The Relationship Between Autistic Traits and Atypical Sensory Functioning in Neurotypical and ASD Adults: A Spectrum Approach. *Journal of Autism and Developmental Disorders*, *47*(2), 316–327. http://doi.org/10.1007/s10803-016-2948-5

Moul, C., Cauchi, A., Hawes, D. J., Brennan, J., & Dadds, M. R. (2015). Differentiating Autism Spectrum Disorder and Overlapping Psychopathology with a Brief Version of the Social Responsiveness Scale. *Child Psychiatry and Human Development*, *46*(1), 108–117. http://doi.org/10.1007/s10578-014-0456-4

Nishiyama, T., Suzuki, M., Adachi, K., Sumi, S., Okada, K., Kishino, H., et al. (2014). Comprehensive comparison of self-administered questionnaires for measuring quantitative autistic traits in adults. *Journal of Autism and Developmental Disorders*, *44*(5), 993–1007. http://doi.org/10.1007/s10803-013-2020-7

Ohl, A., Butler, C., Carney, C., Jarmel, E., Palmieri, M., Pottheiser, D., & Smith, T. (2012). Test–Retest Reliability of the Sensory Profile Caregiver Questionnaire. *American Journal of Occupational Therapy*, *66*(4), 483–487. http://doi.org/10.5014/ajot.2012.003517

Pine, D. S., Guyer, A. E., Goldwin, M., Towbin, K. A., & Leibenluft, E. (2008). Autism Spectrum Disorder Scale Scores in Pediatric Mood and Anxiety Disorders. *Journal of the American Academy of Child and Adolescent Psychiatry*, *47*(6), 652–661. http://doi.org/10.1097/CHI.0b013e31816bffa5

Pugliese, C. E., Kenworthy, L., Bal, V. H., Wallace, G. L., Yerys, B. E., Maddox, B. B., et al. (2015). Replication and Comparison of the Newly Proposed ADOS-2, Module 4 Algorithm in ASD Without ID: A Multi-site Study. *Journal of Autism and Developmental Disorders*, *45*(12), 3919–3931. http://doi.org/10.1007/s10803-015-2586-3

Rutter, M., Bailey, A., & Lord, C. (2003). SCQ. *The Social Communication Questionnaire.* Torrance, CA: Western Psychological Services.

Schoen, S. A., Miller, L. J., Brett-Green, B. A., & Nielsen, D. M. (2009). Physiological and behavioral differences in sensory processing: a comparison of children with autism spectrum disorder and sensory modulation disorder. *Frontiers in Integrative Neuroscience*, *3*, 29. http://doi.org/10.3389/neuro.07.029.2009

Schwarz, G. (1978). Estimating the Dimension of a Model. *The Annals of Statistics*, *6*(2), 461–464. http://doi.org/10.1214/aos/1176344136

Shepherd, B. E., Li, C., & Liu, Q. (2016). Probability-scale residuals for continuous, discrete, and censored data. *Canadian Journal of Statistics*, *44*(4), 463–479. http://doi.org/10.1002/cjs.11302

Shumway, S., Farmer, C., Thurm, A., Joseph, L., Black, D., & Golden, C. (2012). The ADOS Calibrated Severity Score: Relationship to Phenotypic Variables and Stability over Time. *Autism Research*, *5*(4), 267–276. http://doi.org/10.1002/aur.1238

Solomon, M., Olsen, E., Niendam, T., Ragland, J. D., Yoon, J., Minzenberg, M., & Carter, C. S. (2011). From lumping to splitting and back again: Atypical social and language development in individuals with clinical-high-risk for psychosis, first episode schizophrenia, and autism spectrum disorders. *Schizophrenia Research*, *131*(1-3), 146–151. http://doi.org/10.1016/j.schres.2011.03.005

South, M., Carr, A. W., Stephenson, K. G., Maisel, M. E., & Cox, J. C. (2017). Symptom overlap on the SRS-2 adult self-report between adults with ASD and adults with high anxiety. *Autism Research*, *10*(7), 1215–1220. http://doi.org/10.1002/aur.1764

Stewart, C. R., Sanchez, S. S., Grenesko, E. L., Brown, C. M., Chen, C. P., Keehn, B., et al. (2016a). Sensory Symptoms and Processing of Nonverbal Auditory and Visual Stimuli in Children with Autism Spectrum Disorder. *Journal of Autism and Developmental Disorders*, *46*(5), 1590–1601. http://doi.org/10.1007/s10803-015-2367-z

Stewart, E., Cancilliere, M. K., Freeman, J., Wellen, B., Garcia, A., Sapyta, J., & Franklin, M. (2016b). Elevated Autism Spectrum Disorder Traits in Young Children with OCD. *Child Psychiatry and Human Development*, *47*(6), 993–1001. http://doi.org/10.1007/s10578-016-0629-4

Sturm, A., Kuhfeld, M., Kasari, C., & McCracken, J. T. (2017). Development and validation of an item response theory-based Social Responsiveness Scale short form. *Journal of Child Psychology and Psychiatry*, *58*(9), 1053–1061. http://doi.org/10.1111/jcpp.12731

Takarae, Y., Sablich, S. R., White, S. P., & Sweeney, J. A. (2016). Neurophysiological hyperresponsivity to sensory input in autism spectrum disorders. *Journal of Neurodevelopmental Disorders*, *8*(1). http://doi.org/10.1186/s11689-016-9162-9

Takei, R., Matsuo, J., Takahashi, H., Uchiyama, T., Kunugi, H., & Kamio, Y. (2014). Verification of the utility of the social responsiveness scale for adults in non-clinical and clinical adult populations in Japan. *BMC Psychiatry*, *14*(1), 302. http://doi.org/10.1186/s12888-014-0302-z

Tillmann, J., Ashwood, K., Absoud, M., Bölte, S., Bonnet-Brilhault, F., Buitelaar, J. K., et al. (2018). Evaluating Sex and Age Differences in ADI-R and ADOS Scores in a Large European Multi-site Sample of

Individuals with Autism Spectrum Disorder. *Journal of Autism and Developmental Disorders*, *34*(1), 1–16. http://doi.org/10.1007/s10803-018-3510-4

Uljarević, M., Lane, A., Kelly, A., & Leekam, S. (2016). Sensory subtypes and anxiety in older children and adolescents with autism spectrum disorder. *Autism Research*, *9*(10), 1073–1078. http://doi.org/10.1002/aur.1602

Uljarević, M., Prior, M. R., & Leekam, S. R. (2014). First evidence of sensory atypicality in mothers of children with Autism Spectrum Disorder (ASD). *Molecular Autism*, *5*(1), 26. http://doi.org/10.1186/2040-2392-5-26

Wagenmakers, E.-J., & Farrell, S. (2004). AIC model selection using Akaike weights. *Psychonomic Bulletin & Review*, *11*(1), 192–196. http://doi.org/10.3758/BF03206482

Wang, J., Lee, L.-C., Chen, Y.-S., & Hsu, J.-W. (2012). Assessing Autistic Traits in a Taiwan Preschool Population: Cross-Cultural Validation of the Social Responsiveness Scale (SRS). *Journal of Autism and Developmental Disorders*, *42*(11), 2450–2459. http://doi.org/10.1007/s10803-012-1499-7

Wigham, S., McConachie, H., Tandos, J., Le Couteur, A. S., & Gateshead Millennium Study core team. (2012). The reliability and validity of the Social Responsiveness Scale in a UK general child population. *Research in Developmental Disabilities*, *33*(3), 944–950. http://doi.org/10.1016/j.ridd.2011.12.017

Williams, Z. J., Failla, M. D., Gotham, K. O., Woynaroski, T. G., & Cascio, C. (2018). Psychometric Evaluation of the Short Sensory Profile in Youth with Autism Spectrum Disorder. *Journal of Autism and Developmental Disorders*, *48*(12), 4231–4249. http://doi.org/10.1007/s10803-018-3678-7
